# Microbiota-derived short chain fatty acids in pediatric health and diseases: from gut development to neuroprotection

**DOI:** 10.3389/fmicb.2024.1456793

**Published:** 2024-10-08

**Authors:** Chou-Yi Hsu, Lusine G. Khachatryan, Nada Khairi Younis, Mohammed Ahmed Mustafa, Nabeel Ahmad, Zainab H. Athab, Angelina V. Polyanskaya, Elena Victorovna Kasanave, Rasoul Mirzaei, Sajad Karampoor

**Affiliations:** ^1^Thunderbird School of Global Management, Arizona State University Tempe Campus, Phoenix, AZ, United States; ^2^Department of Pediatric Diseases, N. F. Filatov Clinical Institute of Children’s Health, I. M. Sechenov First Moscow State Medical University (Sechenov University), Moscow, Russia; ^3^Department of Pharmacy, Al-Noor University College, Bartella, Iraq; ^4^Department of Medical Laboratory Techniques, University of Imam Jafar Al-Sadiq, College of Technology, Baghdad, Iraq; ^5^Department of Biotechnology and Genetics, Jain (Deemed-to-be) University, Bengaluru, Karnataka, India; ^6^Department of Allied Healthcare and Sciences, Vivekananda Global University, Jaipur, Rajasthan, India; ^7^Department of Biotechnology, School of Allied Sciences, Dev Bhoomi Uttarakhand University Dehradun, Uttarakhand, India; ^8^Department of Pharmacy, Al-Zahrawi University College, Karbala, Iraq; ^9^Venom and Biotherapeutics Molecules Lab, Medical Biotechnology Department, Biotechnology Research Center, Pasteur Institute of Iran, Tehran, Iran; ^10^Gastrointestinal and Liver Diseases Research Center, Iran University of Medical Sciences, Tehran, Iran

**Keywords:** gut barrier function, inflammatory bowel disease, neuroprotection, probiotics, SCFAs

## Abstract

The infant gut microbiota undergoes significant changes during early life, which are essential for immune system maturation, nutrient absorption, and metabolic programming. Among the various microbial metabolites, short-chain fatty acids (SCFAs), primarily acetate, propionate, and butyrate, produced through the fermentation of dietary fibers by gut bacteria, have emerged as critical modulators of host-microbiota interactions. SCFAs serve as energy sources for colonic cells and play pivotal roles in regulating immune responses, maintaining gut barrier integrity, and influencing systemic metabolic pathways. Recent research highlights the potential neuroprotective effects of SCFAs in pediatric populations. Disruptions in gut microbiota composition and SCFA production are increasingly associated with a range of pediatric health issues, including obesity, allergic disorders, inflammatory bowel disease (IBD), and neurodevelopmental disorders. This review synthesizes current knowledge on the role of microbiota-derived SCFAs in pediatric health, emphasizing their contributions from gut development to neuroprotection. It also underscores the need for further research to unravel the precise mechanisms by which SCFAs influence pediatric health and to develop targeted interventions that leverage SCFAs for therapeutic benefits.

## 1 Introduction

The term “microbiota” denotes the vast community of bacteria, fungi, archaea, and viruses inhabiting the human intestinal tract (Mirzaei et al., 2022). The gut microbiota is believed to play a significant role in orchestrating the host’s pathology and physiology (Mirzaei et al., 2022). A wide spectrum of diseases, spanning from localized gastroenterological issues to neurological, respiratory, metabolic, hepatic, and cardiovascular conditions, is closely associated with imbalances in the intestinal microbiota (Cong et al., 2022). One captivating aspect of this field of study is the impact of short-chain fatty acids (SCFAs) produced by the microbiota on the health of children and their susceptibility to various disorders (Zhang et al., 2023). Research in this emerging field suggests that the billions of bacteria residing in the gut play pivotal roles in the overall well-being of newborns and children, extending their influence far beyond the realm of basic digestion (Milani et al., 2017). SCFAs include essential chemical compounds such as acetate, propionate, and butyrate. These molecules have a substantial influence on one’s health and serve as crucial intermediaries that connect the immune, metabolic, and nervous systems, as well as the stomach, within the body (Mansuy-Aubert and Ravussin, 2023).

In the context of immune-mediated diseases, the significance of SCFAs has been extensively evaluated (Chun and Toldi, 2022). For example, insights gleaned from studies involving mouse models have suggested a connection between higher maternal dietary carbohydrates that are accessible to the microbiota, exposure to SCFAs during pregnancy, and a reduced risk of asthma in offspring (Tian et al., 2023; Gray et al., 2017). Notably, human breastmilk samples from mothers with a history of atopy exhibited considerably lower concentrations of acetate and butyrate than did those from nonatopic mothers (Stinson et al., 2020; Prentice et al., 2019). This reduced early-life exposure to SCFAs from human milk could increase the risk of atopic conditions or obesity in breastfed infants (Stinson et al., 2020; Prentice et al., 2019). Furthermore, the research conducted by Pietrzak et al. (2022), which focused on a juvenile population, yielded findings indicating that supplementing with sodium butyrate was ineffective as an additional therapy for children and adolescents recently diagnosed with inflammatory bowel disease (IBD).

Most importantly, recent research has additionally shed light on the potential neuroprotective advantages of SCFAs (Mirzaei et al., 2021). Investigations into various neurodevelopmental and neuropsychiatric disorders in children have sparked considerable interest in the gut-brain axis, a bidirectional communication system connecting the gastrointestinal tract and the brain (Góralczyk-Bińkowska et al., 2022). By functioning as signaling molecules, SCFAs have potential neuroprotective benefits, offering promising avenues for the treatment of conditions such as anxiety, depression, and autism spectrum disorders (ASDs) (Mirzaei et al., 2021).

## 2 The microbiota in fetal and/or neonatal development

Given that the human gut microbiota likely begins developing before birth, environmental exposures during pregnancy can significantly influence the healthy development and composition of the fetal, neonatal, and infant gut microbiota, potentially impacting the health outcomes of the offspring (Coscia et al., 2021; Brown et al., 2013; Stinson et al., 2019). Several investigations underscore the pivotal role of the maternal gut microbiota, both during and after pregnancy, in shaping the enteric energy balance and preventing the development of metabolic syndrome in the postnatal period (Sanidad and Zeng, 2020). While still a subject of debate, emerging data suggest that bacterial colonization in humans may begin as early as the first trimester of pregnancy (Younge et al., 2019; Rackaityte et al., 2020). A recent study by Rackaityte et al. (2020) presented evidence of bacterial seeding, predominantly from *Micrococcaceae* and *Lactobacillaceae*, in the fetal gut. Notably, ex vivo experiments of a live isolate of fetal *Micrococcus* demonstrated the potential to inhibit the generation of interferon-gamma (IFN-γ) by human T cells (Rackaityte et al., 2020).

The concept of in utero bacterial colonization in the fetal intestine aligns with previous discoveries suggesting that the gut harbors memory T cells and dendritic cells (DCs) potentially activated by microbial antigens (Li et al., 2019). Furthermore, a separate study conducted by Aagaard et al. (2014) indicated the presence of bacterial DNA in human placental tissues. In addition, although the location and composition of the gut microbiota in pregnant women differ visibly from those in nonpregnant women, the overall richness and homogeneity of the microbiota do not substantially differ (Qin et al., 2022). However, pregnant women facing difficulties during pregnancy tend to have less diverse gut microbiota, which can be detrimental to both maternal and fetal health. For instance, of the 100 women in the study, 26 had preeclampsia, 25 had aberrant placental growth, 21 were healthy and not pregnant, and 28 were pregnant and in good health (Huang et al., 2021). *Prevotella*, *Porphyromonas*, *Varibaculum*, and *Lactobacillus* were found in significantly lower quantities in preeclamptic women than in pregnant women without this problem. Furthermore, it has been found that infant birth weight is strongly correlated with intestinal microorganisms and, consequently, infant development and growth (Groer et al., 2014).

The microbiota of the offspring is tightly tied to the maternal microbiota throughout pregnancy and during the early years of life, particularly the gut microbiota of newborns and young children (Yao et al., 2021). The perinatal period and the first few years of life are the best times for colonizing a child’s gut microbiota. In the perinatal period, the delivery method and gestational age at birth significantly impact the newborn gut microbiota (Metzler-Zebeli, 2022; Yao et al., 2021). Various factors throughout early life can influence the composition of an infant’s gut microbiota. These factors encompass the technique of feeding, maternal diet, surrounding environment, and host genetic composition. Early life represents a critical period for microbial colonization, and the outcomes of this colonization have substantial implications for newborns’ long-term health and well-being.

## 3 Overview of microbiota-derived SCFAs, production, and signaling

Acetate, propionate, and butyrate are the primary SCFAs produced by specific intestinal microbiota in the cecum and colon (Jasim et al., 2022). Indigestible saccharides that evade digestion in the small intestine, such as dietary fibers, nonstarch polysaccharides, or resistant starch, undergo robust anaerobic glycolysis in the colon, producing SCFAs (Englyst et al., 1992). When there is a scarcity of conventional dietary fibers, amino acids generated by proteolysis can function as alternative substrates for the formation of SCFAs (Cummings and Macfarlane, 1991). Additionally, branched-chain amino acids such as leucine, valine, and isoleucine may undergo fermentation to produce a smaller quantity of minor SCFAs, including formate, valerate, and caproate (Nyangale et al., 2012). SCFAs are synthesized predominantly in the cecum and ascending colon, where they play roles in modulating food absorption, altering the gut pH, supporting immune function, and maintaining overall gastrointestinal health and stability (Blaak et al., 2020; Litvak et al., 2018). The absorption of SCFAs in the colon involves various processes, depending on the hydronation state of the bacteria. Nonionic forms of SCFAs are transported via carrier-mediated mechanisms, while protonated SCFAs are absorbed in the colon epithelium through simple diffusion facilitated by a chemical gradient (Sivaprakasam et al., 2017). The transport of SCFAs involves specific transporters, including sodium-coupled monocarboxylate transporters (MCTs) (SMCT1 and SMCT2), as well as MCT1 and MCT4 (Sivaprakasam et al., 2017). These transporters rely on the chaperone protein CD147 to facilitate their movement to the cell surface (Sivaprakasam et al., 2017). Within the intestinal epithelium, the MCT1, SMCT1, and SMCT2 transporters are expressed on the apical membrane, while MCT1 and MCT4 are expressed on the basolateral membrane (Sivaprakasam et al., 2017). The expression of these transporters may be modulated by several stimuli, including inflammation and SCFAs.

After absorption, SCFAs are directly transported to the liver via the portal vein where they can be converted into endogenous molecules such as cholesterol, fatty acids, and glucose, or function as signaling molecules (Boets et al., 2017). Any unutilized SCFAs are eliminated from the body by exhalation, urination, or defecation (Boets et al., 2017). Notably, only a small amount of SCFAs enter the bloodstream due to rapid and efficient splanchnic extraction (Boets et al., 2017; van der Beek et al., 2015). The liver plays a crucial role in filtering butyrate, removing up to 100% of it while simultaneously supplying approximately 70%–90% of the energy needs of colon epithelial cells (CECs) with very little butyrate reaching systemic circulation (Boets et al., 2017; van der Beek et al., 2015). Nonetheless, circulating SCFAs have been shown to have some impact on cardiometabolic health, suggesting that these effects are dependent on SCFA concentration and receptor interactions (Müller et al., 2019). In addition to their function in supplying energy and nutrients to the colonic mucosa, SCFAs play a pivotal role in the maturation of intestinal epithelial cells (IEC), immunological cells, and adipocytes and hence have significant implications for a range of human diseases (Roediger, 1980; Johnstone, 2002). Two main processes are at play here. First, SCFAs can interact with the transcriptional machinery as histone deacetylase (HDAC) inhibitors (HDACis) (Jasim et al., 2022). Among SCFAs, propionate, which has anti-cancer, and anti-inflammatory properties, closely follows butyrate as the SCFA with the strongest HDACi activity (Donohoe et al., 2012; Frank et al., 2007). Second, SCFAs engage with G protein-coupled receptors (GPCRs) found in various cell types, including IECs, adipocytes, neurons, immune cells, and the vascular endothelium. These GPCRs include G protein-coupled receptor 41 (GPR41) (also known as FFAR3 (Free Fatty Acid Receptor 3)) (specifically responsive to butyrate), GPR43 (also known as FFAR2) (responsive to propionate, butyrate, and acetate), and GPR109A (responsive to butyrate alone). This interaction with GPCRs has wide-ranging effects on cellular and metabolic processes (Donohoe et al., 2012; Frank et al., 2007). In summary, a comprehensive understanding of microbiota-derived SCFAs, from production to signaling, provides valuable insights into their multifaceted roles in maintaining health and preventing diseases.

## 4 Functions of microbiota-derived SCFAs in neonatal health

In addition to their role in the gastrointestinal tract, SCFAs produced by the infant’s intestines can influence various aspects of host physiology in other organs, including the immune system, brain, and adipose tissue (Frost et al., 2014; Koh et al., 2016). In this section, we review and discuss the roles and functions of SCFAs in various aspects of newborn health (Table 1).

**TABLE 1:** Role and action mechanism of microbiota derived short chain fatty acids (SCFAs) on pediatric health.

SCFA	Study setting	Role	Mechanism	Description	References
Acetate, propionate and butyrate	Ex vivo	Permeability, motility and gene expression	FFAR2 TLR2, MCT1, tight-junction and adherens proteins	The SCFAs were found to decrease the permeability of the mucosal layer, perhaps leading to an increase in the absorption of SCFAs. Additionally, the SCFA induced muscular relaxation in the fetal tissue. The aforementioned findings provide support for the notion that SCFA might have a significant impact on prenatal processes and the establishment of imprinting during the latter stages of gestation.	Metzler-Zebeli et al., 2022
Butyrate	In vitro and *in vivo*	Intestinal barrier function	Increase in tight-junction and mucus genes	The induction of an anti-IL-1β response, which is linked to the upregulation of tight junctions and mucus genes in epithelial cells, is attributed to butyrate.	Gao et al., 2021
Acetate, propionate and butyrate	Clinical and in vitro	Infant gut health	Secretory immunoglobulin A (sIgA)	The probiotic formula containing SCFA shown efficacy in preserving the concentration of sIgA, which had experienced a significant decline in infants fed with the placebo formula over a period of time.	Eor et al., 2023
Butyrate	*In vivo*	Digestive functional development	–	Due to the impact of butyrate on enhancing the development of the gastrointestinal (GI) tract and, subsequently, promoting growth performances, this metabolite has shown potential utility in nutrition, as evidenced by studies conducted on calf and piglet subjects.	Kato et al., 2011
Butyrate	*In vivo*	Intestinal development	Glucagon-like peptide-2 (GLP-2)	The inclusion of butyrate in milk replacer shown a good impact on body weight increase, overall health, and some metabolic intermediates in calves. Additionally, it indirectly encouraged rumen development.	Górka et al., 2011
Butyrate	*In vivo*	Intestine development	Villi length and mucosa thickness	Addition of butyrate to the diet may have a positive impact on the growth and maturation of the mucosal lining in the jejunum and ileum of piglets that are given formula.	Kotunia et al., 2004
Acetate, propionate and butyrate	*In vivo*	Induce fetal globin expression	–	SCFAs have the ability to induce the expression of the g globin gene and promote erythropoiesis in an *in vivo* setting.	Pace et al., 2002
Butyrate	Clinical and in vitro	Development of metabolic and immune conditions	Gut microbiota composition	The introduction of supplemental foods at an early stage is linked to changes in the composition of gut microbiota and concentrations of butyrate in stool, which persist until at least one year of age.	Differding et al., 2020
Butyrate	*In vivo*	Expression of inflammatory cytokines	IL-6, IL-8, IFN-γ, IL-10, TGF-β, and histone deacetylase 1 (HDAC1)	Administration of butyrate at an early stage can effectively regulate the inflammatory cytokine levels in the ileum of neonatal piglets. Moreover, this intervention has little effects on the structure of the intestinal microbial community.	Xu et al., 2016
Acetate, propionate and butyrate	Clinical, in vitro	Gut Inflammation	–	There was a significant correlation observed between gut inflammation and fecal SCFAs.	Lefebo et al., 2023
Propionate	*In vivo*	Oxidative stress and inflammation	Nuclear factor erythroid 2-related factor 2 (Nrf2)	The administration of propionate demonstrated a protective effect against lipopolysaccharide (LPS)-induced lung alveolar simplification and aberrant angiogenesis in neonatal rats. This effect was also observed in pulmonary microvascular endothelial cells (HPMECs) and was found to be dependent on the Nrf2.	Chen et al., 2021
Butyrate	*In vivo*	Lipid metabolism	–	Administration of butyrate at an early stage can potentially alter lipid metabolism by reducing the synthesis of fatty acids, as well as influence the profiles of metabolites in the liver.	Yu et al., 2017
Acetate, propionate and butyrate	Clinical and *in vitro*	Infant neurodevelopment	–	Within a population of pregnant women who are in good health, there is a correlation between reduced levels of SCFAs in the bloodstream, particularly during the initial trimester of pregnancy, and improved neurodevelopment in infants.	Hernández-Martínez et al., 2022
Acetate, propionate and butyrate	Cohort and *in vitro*	Growth	–	The exposure of infants to plastic bottles has been found to have an impact on the composition of their gut microbiota at early stages of development, as well as the levels of microbial SCFAs. These alterations in gut microbiota and SCFAs have subsequently been observed to have an effect on the growth of infants.	Zhao et al., 2023
Butyrate	Cross-sectional study and *in vitro*	Growth	–	The quantities of butyrate in human milk exhibited a general negative correlation with both infant weight and adiposity. The concentration of butyrate was shown to be associated with the volume of human milk intake, which suggests a potential mechanism via which butyrate may affect newborn growth by regulating appetite and modulating the intake of human milk.	Olga et al., 2023
Acetate, propionate and butyrate	Clinical	Sleep	–	There is a positive correlation between a greater percentage of total SCFA in fecal samples being composed of propionate and longer periods of unbroken sleep in infants.	Heath et al., 2020
Butyrate	Clinical	Language development	–	The utilization of the all-subsets regression methodology on microbiota data revealed a significant association between the relative abundance of the anaerobic bacterium *Coprococcus eutactus*, known for its butyrate production in the gut, and the development of language skills in infants.	Kort et al., 2021

FFAR2, Free fatty acid receptor 2; *TLR2*, Toll-like receptor 2; *MCT1* monocarboxylate transporter 1; *IFN-γ*, interferon-gamma; *IL*, interleukin; *TGF-β*, transforming growth factor-β.

### 4.1 Gut barrier function

Because of the small quantities of SCFAs present in maternal blood, newborns may be exposed to them through the placenta (Kirschner et al., 2021; Liu et al., 2021). After birth, SCFAs can serve as crucial signaling and energy molecules (Nakatani et al., 2018). Studies involving measurements of SCFA levels in the cecal and abdominal blood of catheterized newborn pigs have suggested efficient paracellular and transcellular absorption of SCFAs (Salminen et al., 1998; Nakatani et al., 2018). However, limited research has been conducted on whether SCFAs may already exert positive effects on nutrition, development, and health during the prenatal period.

As the fetus ingests amniotic fluid and receives oral nutrition, these nutrients interact with the gastric and small intestinal epithelium (Dasgupta et al., 2016). If recognized by the developing fetus, they can trigger receptor-mediated signaling pathways. Therefore, it is imperative to investigate how the fetal small intestine senses SCFAs, especially considering recent evidence from *ex vivo* models demonstrating that the newborn jejunum exhibits significant responsiveness to SCFAs by enhancing muscular contractility (Metzler-Zebeli et al., 2021). The developing gut of piglets, particularly in the final days of gestation, exhibits impairments in intestinal function and barrier integrity similar to those observed in human preterm infants. This similarity makes piglets a valuable model for studying prematurely born human newborns (Ferenc et al., 2014). Recent findings suggest an enhanced barrier function in piglets, which indirectly implies that sodium cotransporters likely facilitate increased SCFA absorption. Interestingly, the fetal jejunal tissue responded differently despite earlier results involving the newborn jejunum that showed enhanced muscular contractility when exposed to the same SCFA solution (Metzler-Zebeli et al., 2021). Instead of increasing contractility, SCFAs cause relaxation of the fetal jejunal muscle tissue, contrary to the initial predictions. These findings highlight the complexity of SCFA interactions and their varying effects in different contexts and developmental stages. Despite the relatively short incubation period, exposure to SCFAs had a noticeable impact on both the functional protein and gene expression levels. These effects included downregulating several SCFA receptors, MCT-1, cytokines, and tight junction proteins.

The reduced transepithelial conductance (GT) induced by SCFAs suggested an enhancement in mucosal barrier function (Metzler-Zebeli et al., 2022). These findings related to GTs imply that tight junction proteins create a physical barrier that restricts the movement of extracellular components from the mucosal side to the serosal side (Metzler-Zebeli et al., 2022).

**FIGURE 1:**
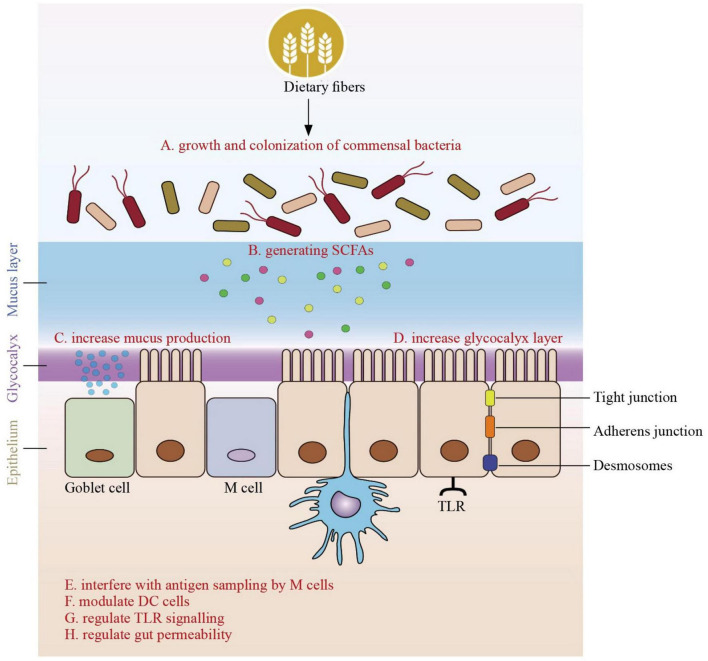
Role of dietary fibers derived short-chain fatty acids on the gut immune barrier in infants. The role of dietary fibers in promoting infant health goes beyond their well-known benefits for digestion. Dietary fiber serves as a vital substrate for the growth and colonization of commensal bacteria in the infant gut. These beneficial microbes ferment dietary fibers, generating short-chain fatty acids (SCFAs) as byproducts. SCFAs, including butyrate, acetate, and propionate, are pivotal in supporting the gut immune system. SCFAs indirectly interact with the gut immune system. They can increase mucus production, which serves as a physical barrier, strengthening the protective shield of the gut. Additionally, dietary fibers enhance the glycocalyx layer, providing additional binding sites for commensal bacteria. This augmentation of the gut’s physical defenses contributes to the overall resilience of the gut immune barrier. Dietary fibers and the SCFAs they produce directly impact immune responses in the infant’s gut. Of note, dendritic cell s (DCs) in the gut act as key antigen-presenting cells, bridging innate and adaptive immunity by sampling antigens from the intestinal lumen and directing the appropriate immune response. In this regard, SCFAs have been shown to affect the maturation state of DC. SCFAs can modulate antigen sampling by affecting the interactions between microfold cells (M cells) and the underlying Peyer’s patches, thus influencing the initiation of immune responses in the gut. Moreover, dietary fibers can modulate dendritic cells, and specialized Toll-like receptors (TLRs) are present around absorptive cells. These interactions help regulate immune responses, ensuring that the immune system responds appropriately to threats while maintaining tolerance to harmless substances. Another critical aspect of the influence of dietary fibers on the gut immune barrier is their role in regulating gut permeability. They help maintain the integrity of the gut lining, preventing the passage of harmful pathogens and antigens into the bloodstream. This regulation is essential for proper immune system functioning and overall health.

In the fetal jejunum, following exposure to SCFAs, the expression of several key components was downregulated. Metzler-Zebeli et al. (2022) investigated the inhibitory effects of SCFAs on HDACs and various receptors, including GPR43, GPR41, hydroxycarboxylic acid receptor 2 (HCAR-2), epidermal growth factor receptor (EGFR), and Toll-like receptor 2 (TLR-2). These receptors have varying affinities for different SCFAs. Consequently, in their study, all the SCFAs most likely engaged with these receptors to some extent. However, when a single SCFA was applied, it resulted in different receptor responses. In the study by Metzler-Zebeli et al. (2022), the combination and concentration of SCFAs largely resulted in the downregulation of FFAR2, TLR2, and EGFR receptor expression rather than the induction of their activation. This finding suggested that the combined action of SCFAs in the mixture had a regulatory effect on these receptors, potentially modulating their function within the fetal jejunum. It is plausible that the high concentration of SCFAs led to downregulation as a negative feedback response to the intense stimulation of active receptors. The activation of pattern recognition receptors such as TLRs, which often transmit signals through the crucial proinflammatory nuclear factor kappa B (NF-κB), is directly linked to the expression of tight junction and adherens junction proteins. The results of the study by Metzler-Zebeli et al. (2022) demonstrated that the fetal jejunum can detect SCFAs during late gestation and initiate physiological responses at both the functional protein level and the gene expression level. This finding suggested that the fetal jejunum can actively respond to SCFAs, potentially fine-tuning its barrier function and immune responses in the presence of these microbial metabolites (Figure 1).

Gao et al. (2021) reported that butyrate potentially downregulates the expression of interleukin (IL)-1-induced genes, namely, chemokine (C-X3-C motif) ligand 1 (*CX3XL1)*, *CXCL5*, and *IL-6*. This finding suggested that butyrate holds promise for mitigating inflammation triggered by IL-1 stimulation within the human small intestine Human 4 cells (H4 cells) (a model system to study the influence of the mucous layer). Additionally, the administration of butyrate to the colon of newborn mice has a noticeable anti-inflammatory effect. Gao et al. (2021) further revealed that butyrate not only counters the depletion of these proteins induced by the IL-1 challenge but also augments the production of claudin-3 and claudin-4. *In vivo*, butyrate also elevates the expression levels of genes associated with tight junctions. Claudins serve as pivotal structural and functional components of tight junctions, effectively impeding the transit of luminal substances through paracellular pathways (Turner, 2009). Given this evidence, Gao et al. (2021) reasonably hypothesized that the increased expression of claudin and occludin proteins may play a pivotal role in mediating the protective effect of butyrate on the intestinal barrier, particularly in mitigating damage induced by IL-1.

Furthermore, their investigation revealed another intriguing aspect of the action of butyrate. It effectively modulates the expression of mucin-related genes both *in vitro* and *in vivo*, suggesting that butyrate exerts control over genes associated with mucin production. The findings of Gao et al. (2021) in E12 cells indicate the presence of a unique mechanism. An increase in butyrate levels did not lead to a significant increase in the expression of the intercellular junction protein zonula occludens 1. Instead, it was predominantly correlated with enhanced mucus generation by goblet cells. To ascertain the anti-IL-1-induced inflammatory effect of butyrate is indeed mediated through the tight junction and mucin signaling pathways in H4 cells, Gao et al. (2021) employed an inhibitor of mucin in their experimental approach. These discoveries underscore the significance of mucin in the butyrate-mediated suppression of the inflammatory response in H4 cells. Based on these findings, microbiota-derived SCFAs play a vital role in shaping pediatric gut barrier function by influencing epithelial integrity and mucus production. Understanding these mechanisms has the potential to guide therapeutic interventions for pediatric gastrointestinal disorders, emphasizing the importance of maintaining a healthy gut microbiota for overall pediatric health.

### 4.2 Immune system

From the earliest stages of life through adulthood, the process of gut microbial colonization in individuals in normal health undergoes a dynamic sequence of events (Zhang et al., 2022). These events play vital roles in maintaining gut equilibrium and initiating the development of a healthy immune system and its corresponding responses (Del Chierico et al., 2015; Dominguez-Bello et al., 2019).

In the newborn gut, microbial signals originating from the gut microbiome are indispensable for the maturation of gut immune cells (Gensollen et al., 2016; Thorburn et al., 2015). This process involves an immunological reaction called the “weaning reaction,” triggered by the expansion of the intestinal microbiota during weaning. During this reaction, T cells transiently produce elevated levels of IFN and tumor necrosis factor (TNF), which can be counteracted by regulatory T cells (Tregs), a type of immune cell fostered by the gut microbiome itself. Moreover, the microbiota in the adult gut, which is primarily composed of *Clostridium* species, contributes to the generation of intestinal Tregs through the production of SCFAs (Al Nabhani et al., 2019; Smith et al., 2013; Furusawa et al., 2013). These microbiota-induced Tregs play a vital role in inhibiting the helper T(Th)-2 response in the context of allergies and the Th)-1/Th17 response in situations such as intestinal inflammation and IBD (Ohnmacht et al., 2015).

Given that bacterial products such as TLR agonists or metabolites such as SCFAs are capable of potentially traversing the placental barrier to reach the fetus, it is plausible that the maternal gut microbiome can influence the development of fetal epithelium and immune cells, even in the absence of live bacterial colonization in the fetal gut (Sanidad and Zeng, 2020). An intriguing study involving genetically modified *E. coli* demonstrated that transient colonization during gestation can increase the population of intestinal ILC3s and F4/80+CD11c+ mononuclear cells in newborns (Gomez de Agüero et al., 2016). Furthermore, pregnant mice fed a high-fiber diet experienced alterations in their gut microbiome and increased SCFA levels. This in-utero exposure led to the suppression of allergic airway disease (AAD) in their offspring, potentially attributable to the elevated levels of SCFAs promoting the development of Tregs (Thorburn et al., 2015). Recent research has highlighted the significance of propionate in prenatal development. Propionate is detected by embryonic IECs via GPCRs, and this interaction promotes the growth of enteroendocrine cells in the fetal intestine (Kimura et al., 2020). The role of butyrate in stabilizing hypoxia-inducible factor 1-alpha (HIF1α) in IECs has significant implications for gut health and permeability. HIF1α plays a crucial role in cellular adaptation to low-oxygen environments, which are characteristic of the gut, particularly the colonic epithelium. Stabilization of HIF1α in IECs can enhance barrier function and promote the integrity of tight junctions, which are essential for preventing gut permeability and “leaky gut.” In the presence of butyrate, studies have shown that HIF1α levels are elevated in IECs, leading to improved barrier function (Kelly et al., 2015). Butyrate achieves this by inhibiting prolyl hydroxylases, the enzymes responsible for HIF1α degradation under normoxic conditions, thus allowing HIF1α to accumulate and exert its protective effects (Kelly et al., 2015). Butyrate’s ability to stabilize HIF1α in IECs has been attributed to multiple mechanisms. First, butyrate serves as a HDAC inhibitor, which alters the transcription of genes involved in maintaining the epithelial barrier and reducing inflammation (Fachi et al., 2019). HDAC inhibition by butyrate enhances HIF1α’s transcriptional activity, leading to the upregulation of genes that support gut barrier integrity, such as those encoding tight junction proteins (e.g., claudins and occludins) (Fachi et al., 2019). Additionally, butyrate’s anti-inflammatory properties mitigate gut inflammation, a major contributor to increased permeability and gut barrier dysfunction. By stabilizing HIF1α, butyrate not only strengthens the intestinal barrier but also reduces gut permeability, which is critical for preventing systemic inflammation and maintaining overall gut health (Fachi et al., 2019). Increased gut permeability is a hallmark of various gastrointestinal disorders, such as IBD, and butyrate’s role in promoting HIF1α stabilization presents a therapeutic target for these conditions.

These findings collectively underscore the profound influence of maternal gut microbiome-derived SCFAs on both prenatal and postnatal development, with potential implications for maintaining enteric energy balance and preventing postnatal metabolic syndrome. These findings shed light on how the stimulation of in-utero microbial activity, likely originating from the maternal gut microbiome, can modulate the development of both the immune system and intestinal cells during both the prenatal and postnatal phases.

In a study led by Pace et al. (2002), the focus shifted toward SCFA derivatives, intending to determine *in vivo* plasma concentrations sufficient to induce fetal globin production. Oral or intravenous doses of sodium phenylbutyrate and arginine butyrate were administered to the baboons, corresponding to 8% to 20% of the required effective doses needed to achieve plasma concentrations of SCFA derivatives above the targeted levels. Remarkably, a 5- to 10-milliliter dose of either compound, when administered to an adult of average weight, could sustain plasma concentrations up to 20 times higher than the desired threshold levels for several hours (Pace et al., 2002). This efficacy was made possible because the sodium salts of the two primary derivative compounds, sodium α-methyl hydrocinnamate and sodium 2,2-dimethyl butyrate, can be readily formulated into aqueous solutions. These results demonstrated that specific SCFA derivatives (SCFADs) play a role in inducing the expression of gamma-globin *in vivo*, both in animals with normal erythropoiesis and in anemic baboons, whose metabolic clearance rates are more akin to, though still greater than, those observed in humans. Across all animal models studied, three of these derivatives exhibited a significant increase in gamma-globin gene expression even before any erythropoietic effects occurred, comparable to the impact observed with butyrate (McDonagh et al., 1992; Blau et al., 1993; Pace et al., 1994). In the anemic baboon model, the level of F-reticulocyte induction by these derivatives was comparable to the level of gamma-globin in baboons previously treated with significantly greater doses of more rapidly metabolized SCFAs, such as butyrate, acetate, propionate, and pentanoic acid (Blau et al., 1993; Stamatoyannopoulos et al., 1994). Notably, the baboons in Pace et al. (2002) study exhibited a response to SCFADs at remarkably low single doses of just 50 mg/kg, whether administered parenterally once daily or orally every other day, three days per week. This is in contrast to longer-chain fatty acids, which require much higher concentrations of fatty acids *in vitro* or doses ranging from 1000 to 4000 mg/kg in primate studies (McDonagh et al., 1992; Pace et al., 1994; Constantoulakis et al., 1988; Constantoulakis et al., 1989; Liakopoulou et al., 1995). In conclusion, these findings strongly suggest that at least two of the investigated SCFADs hold significant promise as potential treatments for stimulating gamma globin production in patients with beta hemoglobinopathies. These SCFADs can activate F cells and promote erythroid proliferation at considerably lower dosage requirements than currently utilized drugs such as butyrate and phenylbutyrate. However, further investigations are necessary to understand the complete impact of SCFAs on immune cell subsets in neonates.

### 4.3 Brain and behavior

The interconnected gut-brain axis is garnering increasing attention, with SCFAs previously associated with regulating neuroimmune and neuroendocrine functions (Silva Y. P. et al., 2020). A network of neurons that oversees intestinal mucosal activities is intricately linked to the body’s immune response mediated by immunological effector molecules (Chesné et al., 2019). SCFAs, when transported through the stomach, may directly influence vagal afferent nerves, which play crucial roles in regulating satiety, stress responses, and mood (Silva Y. P. et al., 2020). Moreover, microbiota SCFAs may cross the blood-brain barrier (BBB) and enter the bloodstream and cerebrospinal fluid (CSF) within the brain (Vijay and Morris, 2014; Bachmann et al., 1979). This potential transport mechanism could directly impact the levels of neurotrophic factors, which govern the development and differentiation of synapses and neurons within the brain.

A growing body of evidence strongly suggests that the colonization of the neonatal intestine by microbes plays a pivotal role in priming the immune system, establishing communication with the brain through the afferent vagus nerve, and generating SCFAs that can directly or indirectly influence brain function (Luck et al., 2020). Indeed, research focusing on SCFAs during pregnancy and during the initial years of a child’s life reveals that SCFAs originating from the mother can traverse the placenta (Hernández-Martínez et al., 2022). This exposure occurs at critical developmental stages and significantly influences essential neurodevelopmental processes, such as cellular differentiation, neurosphere formation, the transformation of embryonic stem cells into neural cells, neural proliferation, and the maturation and functioning of microglia (Carlson et al., 2018; Mirzaei et al., 2021; Yang et al., 2020).

Recent research highlights that SCFAs, such as butyrate, promote the production of brain-derived neurotrophic factor (BDNF), facilitate neurogenesis, and contribute to the consolidation of long-term memory (Yang et al., 2020; Yoo et al., 2011; Kim et al., 2009; Wei et al., 2014; Levenson et al., 2004). Although propionate is the least studied SCFA, it is noteworthy that it still has positive health effects and is important for brain growth, cognition, and behavior (Yang et al., 2020; Ziętek et al., 2021; Hsiao et al., 2013). Animal studies have provided valuable insights into the impact of prenatal dietary factors on offspring behavior and neurodevelopment. Research indicates that a low-fiber diet during pregnancy is associated with reduced levels of propionate and butyrate. Moreover, offspring from mothers on a low-fiber diet tend to exhibit diminished overall locomotor activity and an increased prevalence of anxiety-related behaviors (Yu et al., 2020). These findings suggest that a reduction in propionate and butyrate levels due to a low-fiber diet during pregnancy may have adverse effects on offspring. However, other animal studies have presented a different perspective. Mice that received exogenous injections of propionate during pregnancy exhibited adverse effects, including impaired memory and learning, altered locomotion, and the development of stereotyped social and anxiety-related behaviors (Foley et al., 2014; Foley et al., 2015; Nankova et al., 2014). This finding underscores the complexity of the relationship between SCFAs such as propionate and behavioral outcomes and suggests that maintaining an appropriate balance is crucial for optimal neurodevelopment. Indeed, both low and high SCFA levels may have an impact on neurodevelopmental processes. Despite animal studies and research involving prenatal SCFA administration or dietary modification during pregnancy, it remains unclear whether there is a direct connection between circulating serum SCFA levels during pregnancy and neurodevelopment as well as infant behavior, both in animals and humans (Yang et al., 2020; Diaz Heijtz et al., 2011). Hernández-Martínez et al. (2022) found evidences consistent with prior research showing that the offspring of pregnant dams with elevated propionate levels exhibit altered locomotor activity and increased anxiety-related behaviors (Yang et al., 2020; Foley et al., 2014; MacFabe, 2015). These results contrast with those reported by Dawson et al. (2021), who reported that mothers of children with lower emotionality scores at age two had higher levels of specific butyrate-producing organisms in the third trimester, even though direct measurements of butyrate levels were not taken. Although an excess of SCFAs may have harmful effects, there is a consensus in the scientific community that SCFAs generally positively influence brain development (Serino, 2019).

Magzal et al. (2021) observed a striking connection between a greater proportion of propionate in the fecal SCFA composition and longer periods of uninterrupted infant sleep. Even a 1% increase in propionate was associated with a six-minute extension in the duration of the infant’s longest uninterrupted sleep overnight. This finding suggested the intriguing possibility of enhanced sleep efficiency, potentially contributing to improved sleep state organization.

Kort et al. (2021) have also delved into the connection between gut microbiota composition and language development in children. One of the most significant findings of the current study pertains to the link between the presence of saccharolytic *Clostridia* members in children’s guts at 24 months of age and the composite score reflecting language development in those same children at 36 months. This association included recognizing two specific species, *Coprococcus eutactus* and *Intestinibacter bartlettii*. Both of these species are known to produce SCFAs. Studies conducted with animal models have provided valuable insights into the potential benefits of butyrate in addressing the adverse effects of stress on neurotrophic factors and cognitive function (Kort et al., 2021). These findings are consistent with the notion that the production of SCFA butyrate in the gut may promote improved language development, as it may have positive effects on brain function and development (Valvassori et al., 2014). In summary, the evolving field of research on microbiota-derived SCFAs indicates their pivotal role in influencing brain development, behavior, and various neurodevelopmental processes.

### 4.4 Growth

The capacity of the gut microbiota to produce SCFAs and essential vitamins plays a critical role in supporting the metabolic processes necessary for achieving optimal physical growth and development (Kane et al., 2015). Olga et al. (2023) revealed a general inverse correlation between human milk-derived butyrate concentrations and various infant weight and adiposity indicators. This finding suggested that butyrate in human milk may play a role in mitigating the risk of childhood obesity and excessive weight gain. Research in both humans and animals has linked butyrate and the bacteria responsible for its production to a reduced likelihood of obesity and metabolic issues such as liver fibrosis and insulin resistance (Arnoldussen et al., 2017; Pannaraj et al., 2017; Lin et al., 2012). Furthermore, in a piglet model, butyrate appeared to influence lipid metabolism by enhancing adipogenesis and fat accumulation (Yan and Ajuwon, 2015). These findings underscore the potential impact of butyrate in human milk on infant metabolic health and weight regulation.

Interestingly, the negative relationships between butyrate intake and early infant development were less pronounced when considering butyrate intake through human milk than when considering butyrate concentrations (Olga et al., 2023). This could be because the human milk butyrate concentration and the volume of milk intake exhibited an inverse relationship. Specifically, the high butyrate concentration in human milk might have contributed to some infants having lower human milk intake, potentially influencing their growth patterns (Olga et al., 2023). Recent animal studies have demonstrated that acute oral administration of butyrate through intragastric gavage can swiftly induce feelings of satiety and reduce food intake in mice (Jin et al., 2015; Yadav et al., 2013). This effect is likely mediated through the activation of neuropeptide XY neurons via vagal nerves (Li et al., 2018). Furthermore, other SCFAs, such as propionate, have been recognized as significant regulators of the gut-brain axis signaling pathway and appetite control (Li et al., 2017). In light of these findings, it is conceivable that the interaction between the odor and/or taste of butyrate in human milk and its potential impact on appetite regulation could reduce human milk intake in newborns.

In a study conducted by Kotunia et al. (2004), mice supplemented with Na-butyrate exhibited increased final body weights. However, this finding should be interpreted with caution because the animals in the experimental group initially gained more body weight. This discrepancy in starting body weight gain could influence the final outcomes.

Studies have demonstrated that butyrate can directly stimulate epithelial cell proliferation in organ cultures of human colonic mucosa, even in the absence of circulating neural factors (Scheppach et al., 1992). However, the explanations for the alterations observed in the distal ileum and jejunum are complex. In the case of the distal small intestine, oral intake of Na-butyrate appears to promote its development indirectly rather than through a direct mechanism, although the exact underlying mechanism remains unknown. Interestingly, research has shown that the unexposed epithelium of the ileum and jejunum can still experience substantial systemic trophic and mitotic effects when butyrate is introduced into the colon (Sakata, 1987; Frankel et al., 1994). This finding suggested that butyrate may have systemic effects that extend beyond direct contact with the local intestinal mucosa, impacting regions of the gut that are not in direct contact with the compound. However, the exact mechanisms involved in this phenomenon require further investigation.

In experiments with rats, isolated and denervated jejunal loops were induced to develop through SCFA infusions into the colon lumen (Sakata, 1989). Interestingly, the trophic effects of other SCFAs were observed primarily on the colonic mucosa when the SCFAs were infused directly into the colon, suggesting that this trophic action might be unique to butyrate. Consequently, the trophic effects of other SCFAs on the colon appear to be locally mediated (Frankel et al., 1994). Kotunia et al. (2004) showed that Na-butyrate has a nutritional effect on smooth muscles located in the duodenum, proximal jejunum and ileum. This finding indicates that hypertrophy may not be limited to the gut tunica mucosa but could also involve changes in the smooth muscle layer of the intestine. Overall, the observed modifications in the SCFA-rich guts seem to contribute to improved feed consumption and greater body mass growth. These changes reflect the broader impact of SCFAs on the gut’s structure and function, ultimately influencing newborns’ overall growth and development.

### 4.5 Metabolic function

Numerous recent studies have shown that SCFAs can enhance the differentiation of adipocytes and induce metabolic adjustments in fat cells (Li et al., 2014). This includes the upregulation of genes responsible for fatty acid oxidation and the downregulation of genes involved in fatty acid synthesis (Li et al., 2014). The distribution of fat in adipose tissue, particularly factors such as backfat thickness and intramuscular fat concentration, plays a crucial role in determining pork flavor. However, it has not been conclusively established whether SCFAs may function as distinctive regulators of lipid metabolism in various tissues. Yu et al. (2017) examined the effects of early sodium butyrate intervention on newborn pigs’ lipid metabolism and liver metabolite profiles. Compared to those in the control group, the findings revealed that sodium butyrate treatment induced notable changes in the expression of genes related to adipose tissue differentiation and lipid metabolism, as well as alterations in the liver metabolite profiles. This investigation highlights the significant influence of early sodium butyrate intervention on lipid metabolism. Specifically, Yu et al. (2017) reported that treatment with sodium butyrate led to a substantial increase in the expression of adipocyte markers such as leptin, fatty acid binding protein 4 (FABP4), and peroxisome proliferator-activated receptor (PPAR) in adipose tissue by day seven. These results suggest that sodium butyrate may play a role in promoting adipocyte differentiation in newborn pigs. These findings align with previous research conducted by Li et al. (2014), who reported that butyrate stimulates the expression of sterol regulatory element-binding transcription factor 1c (SREBP-1c), transcription factor CCAAT/enhancer binding protein (C/EBP), and PPAR in the stromal vascular fraction of adipose tissue in pigs. Additionally, Yan and Ajuwon (2015) reported a noticeable upregulation in the expression of PPAR and C/EBP in adipocytes treated with butyrate. This observation is consistent with the findings of Fajas et al. (1999) and Kim et al. (1998), which suggested that an increase in SREBP-1c expression may enhance PPAR expression. Notably, the results of Yu et al. (2017) also indicated that, compared with control treatment, sodium butyrate therapy increased SREBP-1c expression in adipose tissue, further supporting this relationship. Furthermore, research by Rumberger et al. (2014) demonstrated that butyrate and other SCFAs expedite the process of lipolysis in 3T3-L1 adipocytes (a sub-clonal cell line derived from the original 3T3 Swiss albino cell line), adding to the body of evidence regarding the impact of butyrate on lipid metabolism. According to the research conducted by Yan and Ajuwon (2015), butyrate exerts direct effects on adipocytes, influencing the control of lipid storage by preventing lipolysis and promoting fatty acid synthesis in stromovascular cells. Specifically, they noticed a substantial decrease in the expression of lipogenic genes, including *ACC*, *PPAR*, and *CPT-1a*, following the administration of sodium butyrate therapy in comparison to the control treatment.

Previous studies by Fang et al. (2014) and Sun et al. (2016) demonstrated that a highly resistant starch diet can lead to reduced levels of cholesterol and triglycerides in the blood or liver of pigs by increasing the production of SCFAs, notably butyrate, in the large intestine. Similar effects have been observed in other animal models. For instance, Hara et al. (1999) found that SCFAs, including butyrate, slowed cholesterol production and decreased serum cholesterol levels in the livers of rats. Gao et al. (2009) also reported that butyrate lowered cholesterol and triglyceride levels in mice fed with a high-fat diet. However, it should be noted that while sodium butyrate significantly impacted cholesterol metabolism, it did not appear to affect lipogenesis in the liver. A study conducted by Yu et al. (2017) employed gas chromatography-mass spectrometry (GC–MS) analysis to investigate the effects of sodium butyrate intervention on several processes associated with carbohydrate metabolism, including glycolysis, gluconeogenesis, and nucleotide sugar metabolism. The results revealed substantial impacts of the intervention on these pathways. Building upon earlier research by Samuel et al. (2008) and Xiong et al. (2004), it is important to note that butyrate serves not only as an energy source for animals but also as a signaling molecule that plays a pivotal role in regulating the body’s energy metabolism. In the study by Yu et al. (2017), the administration of sodium butyrate led to notable changes in metabolite levels. Specifically, they observed higher levels of glucose-6-phosphate and lower levels of pyruvic acid. These findings suggest that sodium butyrate may enhance the capacity for glucose phosphorylation while inhibiting glycolysis in the liver. Furthermore, Yu et al. (2017) revealed that sodium butyrate therapy led to a reduction in the levels of glyceric acid and glyceric acid-3-phosphate. This observation suggested that, compared with those in the control group, the pigs receiving sodium butyrate treatment may have had less fat deposited in their livers. Consequently, early intervention with sodium butyrate appears to modulate hepatic lipid metabolism by reducing the production of fatty acids, consistent with the findings of the gene expression study. In conclusion, microbiota-derived SCFAs represent a fascinating area of research for understanding the intricate interplay between the gut microbiota and pediatric metabolic function. As the scientific community continues to unravel the complexities of this relationship, there is potential for novel strategies to optimize metabolic health in children and mitigate the risk of metabolic disorders later in life.

## 5 Mechanistic involvement of short-chain fatty acids in the development and prevention of neonatal diseases

As reviewed above, SCFAs play multifaceted roles in neonatal health, ranging from shaping the gut microbiota to regulating the immune system and enhancing barrier function. Additionally, the therapeutic potential of SCFAs opens new avenues for neonatal healthcare, offering promising strategies for disease prevention and improved neonatal health outcomes. In this section, we provide an overview and discuss the mechanistic roles of SCFAs in the progression and prevention of neonatal diseases (Table 2).

**TABLE 2:** Function of microbiota derived short chain fatty acids (SCFAs) in pediatric diseases.

Disease	Study setting	SCFA	Role	Description	References
Food allergy	Clinical	Butyrate	Tolerance via influencing the bacterial community structure	The promotion of tolerance in newborns with cow’s milk allergy was observed with the administration of extensively hydrolyzed casein formula supplemented with *Lactobacillus rhamnosus GG* (EHCF + LGG). This intervention was found to exert an influence on the bacterial community structure at the strain level within the gastrointestinal tract of the infants.	Berni Canani et al., 2016
Food allergy	Clinical	Butyrate	Non-IgE-mediated and IgE-mediated	The prevalence of *Bacteroides* was found to be greater in children diagnosed with IgE-mediated Cow’s milk allergy (CMA) in comparison to individuals with non-IgE-mediated CMA and healthy individuals. *Bacteroides* may play a significant role in the development of CMA and suggests the existence of shared mechanisms that contribute to both non-IgE- and IgE-mediated food allergies.	Berni Canani et al., 2018
Food allergy	Clinical and *in vivo*	Butyrate	Immunoregulatory effects	The administration of butyrate prior to allergen exposure resulted in a notable decrease in the allergic reaction. This effect was shown through the suppression of T helper 2 cytokine production and the promotion of tolerogenic cytokines. The study found the activation of human beta defensin-3 and upregulation of tight junctions in human enterocytes upon exposure to butyrate in peripheral blood mononuclear cells derived from children with food allergy. Additionally, the expression of interleukin 10, interferon gamma, and forkhead box P3 (FOXP3) was found to be modulated through epigenetic mechanisms.	Paparo et al., 2021
Food allergy	Clinical	Butyrate	Alterations in the composition of fecal microbiota	The fecal concentration of total SCFAs was shown to be comparatively lower in children with cow milk protein allergy as compared to their healthy counterparts.	Dong et al., 2018
Atopic dermatitis (eczema)	Clinical	Butyrate	Gut microbiota dysbiosis	The study has discovered a potential association between microbial activity and the development of eczema. This finding suggests that there may be a less than ideal establishment of gut microbiota at certain developmental periods in newborns who are at a heightened risk for allergies.	Wopereis et al., 2018
Eczema	Clinical	Propionate	Gut microbiota dysbiosis	Reduced levels of SCFAs, succinate, phenylalanine, and alanine were observed in fecal samples obtained from children who subsequently developed dermatitis.	Kim et al., 2015
Eczema	Clinical	Iso-butyric, iso- valeric acid and valeric acid	–	At the age of 3, children living on farms exhibited elevated amounts of iso-butyrate, iso-valeric acid, and valeric acid in comparison to their counterparts residing in rural areas. Furthermore, children who had older siblings exhibited elevated levels of valeric acid by the time they reached 3 years of age. There was a significant correlation observed between elevated concentrations of valeric acid at the age of three and a decreased incidence of eczema at the age of eight.	Kim et al., 2015
Asthma	Cohort study and *in vivo*	Propionate	GPR41	The ingestion of propionate via breast milk during the lactation phase led to a notable decrease in airway inflammation in the offspring in a mouse model of asthma generated by house dust mites.	Ito et al., 2023
Atopy	Clinical	Butyrate	Immune modulation	The newborns who later developed allergy sensitization in infancy exhibited a deficiency in genes responsible for encoding crucial enzymes involved in carbohydrate breakdown and butyrate synthesis within their microbiome.	Cait et al., 2019
Overweight and atopy	Clinical	Propionate	IgA	The child outcomes were found to be influenced by the colonization of *Clostridium difficile* at 3 months, with metabolites propionate and formate serving as secondary pathways. Notably, formate was identified as a crucial factor that intersected many pathways.	Vu et al., 2021
Obesity	Clinical	Butyrate	–	The content of butyrate in stool exhibited a positive correlation with the prevalence of child weight outcomes, suggesting the need for additional investigation into its potential role as a contributing factor in the development of childhood obesity.	Nandy et al., 2022
Obesity	Clinical	Butyrate	–	The administration of oral butyrate supplements demonstrated efficacy in the management of pediatric obesity.	Coppola et al., 2022
Obesity	*In vitro*	Acetate, propionate and butyrate	–	Prebiotic supplements may vary in their capacity to promote SCFA generation in children and adolescents who have obesity.	Holmes et al., 2020
Obesity	*In vivo*	Acetate	–	The supplementation of infant milk formula with heat-treated probiotic *Bifidobacterium animalis subsp. lactis* CECT 8145 (referred to as HT-BPL1-IN) resulted in a considerable reduction in fat accumulation in the model organism *Caenorhabditis elegans*. Concurrently, this supplementation led to a substantial increase in the production of acetate and the organic acid lactate.	Silva Á. et al., 2020
Obesity	Cohort	Acetate, propionate and butyrate		SCFAs found in human milk have a positive impact on weight gain and obesity in infants.	Prentice et al., 2019
Liver steatosis	*In vivo*	Butyrate	AKT/AKT and GPX1	The administration of butyrate throughout the prenatal period resulted in a reduction in the severity of inflammation, oxidative stress, and apoptosis in both the maternal and fetal subjects.	Huang et al., 2022
Celiac disease	Clinical	Acetate and caproic acid	–	The analysis of SCFAs revealed a significant decrease in acetate concentration and a modest increase in caproic acid concentration in the group with celiac disease.	Seničar et al., 2021
Prenatal stress	*In vivo*	Butyrate	Cognitive function	Butyrate had a neuroprotective effect, attributed to the upregulation of neural growth factor, resulting in enhanced cognitive function among infants who experienced prenatal stress.	Akrami and Edalatmanesh, 2016
Neonatal hypoxia-ischemia	*In vivo*	Butyrate	Neuroprotection and neurogenesis	The administration of butyrate resulted in a significant decrease in cerebral injury induced by hypoxic-ischemic conditions. Additionally, the utilization of this histone deacetylase inhibitor (HDACi) demonstrated a protective effect against the loss of neuroblasts and oligodendrocyte precursor cells induced by hypoxic-ischemic conditions, as well as against neuroinflammation.	Ziemka-Nalecz et al., 2017
Neonatal hypoxia-ischemia	*In vivo*	Butyrate	CXCL10, IL-1β, COX-2, and inflammation	The administration of butyrate resulted in a decrease in brain damage, inhibition of the synthesis of inflammatory markers such as IL-1β and chemokine CXCL10, and prevented the overexpression of COX-2 in the injured hemisphere affected by ischemia. In addition, the administration of butyrate facilitated the transition of microglia phenotypic from an inflammatory M1 state to an anti-inflammatory M2 state.	Jaworska et al., 2017
Ulcerative colitis (UC)	Cross-sectional study	Acetate, butyrate, iso-valeric acid, and propionate	–	The amounts of acetate, butyrate, iso-valeric acid, and propionate were shown to be significantly lower in children diagnosed with UC when compared to individuals of the same age, gender, and with similar disease activity.	Rotondo-Trivette et al., 2021
Stunted children	Clinical and *in vitro*	Acetate and valerate	TNF-α, IL-10, LPS binding protein and secretory IgA	This study found the associations among the gut microbiota composition, immune response status, and stool SCFAs concentrations in children with stunted growth compared to children of the same age with normal nutritional status.	Surono et al., 2021
Necrotizing enterocolitis (NEC)	*In vitro*	Acetate, propionate and butyrate	G-protein coupled receptor (GPCR)	The study has identified a mechanism that elucidates the synbiotic effect resulting from the interaction between expressed breast milk and probiotics administered to preterm infants, so effectively mitigating excessive inflammation and reducing the risk of necrotizing enterocolitis. The study additionally posits that the anti-inflammatory impact of SCFAs in the developing intestine varies from the mechanism observed in the fully developed colon.	Zheng et al., 2020
NEC	Clinical	Acetate, propionate and butyrate	–	The observed rise in *Streptococcus salivarius* and *Rothia mucilaginosa*, along with the concurrent decline in *Bifidobacterium_animals_subsp._lactis*, as well as the reduction in acetate, propionate, and butyrate, may potentially serve as valuable indicators for the early detection of NEC.	Liu et al., 2022
NEC	*In vivo*	Butyrate	–	The lack of distinct biological indicators for NEC necessitates the reliance on the data presented, which offers novel and valuable mechanistic understandings of the underlying pathophysiology of the disease. This knowledge is crucial in the pursuit of generating innovative therapeutic interventions.	Ferraris et al., 2023
NEC	*In vivo*	Butyrate	HMGB1, TLR4, NF-κB, IL-1β, IL-6, IL-8, and TNF-α	The administration of butyrate alleviated intestinal inflammation and partially restored the dysregulated intestinal microbiota in mice afflicted with NEC.	Sun et al., 2021
NEC and food protein-induced allergic protocolitis (FPIAP)	Clinical	Acetate, propionate and butyrate	–	The potential biomarker targets for early detection of NEC and FPIAP could include variations in gut microbiota composition and concentrations of SCFAs.	Xiong et al., 2022
Congenital Chloride Diarrhea (CLD)	Clinical	Butyrate	–	The administration of butyrate therapy resulted in sustained and secure management of diarrhea severity in individuals with CLD.	Di Meglio et al., 2021
CLD	Clinical	Butyrate	–	The findings revealed that the effectiveness of butyrate as a therapeutic intervention in CLD was influenced by the specific genotype of the individual.	Canani et al., 2013
Respiratory infection	*In vivo*	Propionate	Flt3L	The administration of therapy involving a specific strain of bacteria that produces propionate, which was obtained from the milk of mothers who followed a high-fiber diet, or the addition of propionate itself, had a protective effect against severe lower respiratory infections (sLRI). This protection was achieved by restoring the expression of Flt3L in the gut and promoting the production of plasmacytoid dendritic cells (pDCs) involved in hematopoiesis.	Sikder et al., 2023
Respiratory syncytial virus (RSV) bronchiolitis	*Ex-vivo*	Acetate	RIG-I	The application of acetate to respiratory cells obtained from patients in an *ex-vivo* setting resulted in a decrease in RSV load. Additionally, this therapy led to an increase in the expression of interferon-stimulated genes (ISGs) OAS1 and ISG15, as well as the viral recognition receptors mitochondrial antiviral-signaling protein (MAVS) and RIG-I.	Antunes et al., 2022
Bronchopulmonary dysplasia (BPD)	*In vivo*	Acetate	NLRP3 Inflammasome	Following the administration of acetate, there was a notable decrease in the expression levels of TNF-α, IL-1β, IL-18, NLRP3, and caspase-1. Conversely, the expression of GPR43 exhibited a large rise. In the BPD mice who received acetate treatment, there was a decrease in the proportion of Escherichia-Shigella compared to the BPD mice that received a placebo. Additionally, the abundance of *Ruminococcus* was found to be higher in the acetate-treated BPD animals.	Zhang et al., 2021
Preterm neonates	Clinical	Acetate	-	The use of acetate in neonatal parenteral nutrition has been found to effectively mitigate the occurrence of metabolic acidosis and hyperchloraemia.	Peters et al., 1997

*Ig*, immunoglobulin; GPR41, G protein-coupled receptor 41; *AKT* protein kinase B; GPX1 Glutathione peroxidase 1; *CXCL10*, CXC chemokine ligand 10, IL; COX-2 *TNF-α*, tumor necrosis factor α; *IL*, interleukin; *LPS*, lipopolysaccharide; *HMGB1*, high mobility group box 1; *TLR* Toll-like receptor; *Flt3L*, Fms-like tyrosine kinase 3 ligand; *RIG-I*, retinoic acid-inducible gene I; *NLRP3*, pyrin domain-containing protein 3.

### 5.1 Anti-inflammatory activity

Among the numerous bioactive metabolites the gut microbiota produces, SCFAs have garnered significant attention for their potential role in fostering anti-inflammatory activity (Jasim et al., 2022). According to previous reports, propionate plays a pivotal role in regulating lung and airway inflammation (Chen et al., 2021). For instance, treatment with propionate has been demonstrated to reduce allergic lung irritation triggered by household dust mites (Chen et al., 2021). In studies involving mice, those treated with propionate exhibited protection against the development of allergic airway inflammation. Sodium propionate may promote the polarization of Alternatively activated macrophages (M2 macrophages) while inhibiting Classically activated macrophages (M1 macrophages) (Chen et al., 2021). This finding indicates the potential of this chemical to influence the balance of immune responses. Furthermore, research has suggested that propionate levels can modulate immune responses in the lungs both *in vivo* and *in vitro* (Chen et al., 2021). There is also a connection between increased propionate production by the gut microbiota and reduced levels of lung inflammation. This highlights the intricate relationship between gut health and lung health. Inflammation and angiogenesis are key characteristics associated with the transcription factor NF-κB, which serves as a master regulator of the inflammatory response (Chen et al., 2021). Therefore, the role of propionate in regulating NF-κB activity could have significant implications for managing inflammatory processes in the lungs and airways.

Chen et al. (2021) reported that the use of propionate in human pulmonary microvascular endothelial cells (HPMECs) successfully inhibited the lipopolysaccharide (LPS)-induced increase in p65 phosphorylation and the subsequent nuclear translocation of p65 NF-κB. Notably, proinflammatory factors such as IL-1, TNF-α, IL-6, and IL-8, which are significant biomarkers associated with predicting adverse pulmonary outcomes in preterm infants, were found to be expressed at elevated levels in the mRNA of newborn mice and HPMECs exposed to LPS. These pro-inflammatory factors are linked to the activation of NF-κB. In summary, Chen et al. (2021) suggested that the activation of NF-κB is a key component in the development of LPS-induced bronchopulmonary dysplasia (BPD) and that the inhibition of NF-κB activation by propionate treatment in HPMECs is associated with a reduction in proinflammatory factors that are known to be detrimental to the pulmonary health of preterm infants. Preterm newborns, due to the transition from intrauterine hypoxia to extrauterine hyperoxia, are particularly vulnerable to oxidative stress and have a greater risk of developing BPD. The implementation of antioxidant treatments holds promise for preventing BPD. Chen et al. (2021) found that propionate treatment effectively reduced the alveolar simplification caused by LPS exposure. Furthermore, propionate treatment led to an increase in the mRNA levels of antioxidant genes, such as superoxide dismutase (*SOD*) 1, *SOD2*, *Gclm*, and *Txn*, as well as enhanced SOD activity in both lung and serum tissues *in vivo*. Additionally, propionate treatment decreased the production of reactive oxygen species (ROS) and reduced the mRNA levels of antioxidant genes in HPMECs (Chen et al., 2021). These findings collectively suggest that propionate treatment may mitigate the development of BPD by reducing inflammation, enhancing antioxidant defenses, and mitigating oxidative stress, particularly in the context of preterm infants exposed to changes in oxygen levels during early development.

Xu et al. (2016) investigated the impact of early butyrate intervention on the gut microbiome’s composition and the production of inflammatory cytokines in newborn pigs. These findings revealed that butyrate therapy enhanced the diversity of the microbiota in the stomach and led to alterations in the gene expression of inflammatory cytokines in the ileum. Additionally, this intervention influenced the gene expression of inflammatory cytokines. Butyrate has shown promise in experimental trials for its anti-inflammatory effects in IBD (Couto et al., 2020; Li G. et al., 2021). Additionally, supplementing with oral butyrate has been found to mitigate dysbiosis in patients with ulcerative colitis (UC) (Vieira et al., 2012). However, Pietrzak et al. (2022) reported that sodium butyrate supplementation was not effective as an adjunct therapy for newly diagnosed IBD in children and teenagers. Despite the fact that the current study involved a smaller group of adult patients with Crohn’s disease (CD) and UC, the findings align with those of other studies (Facchin et al., 2020). These consistent results suggest that sodium butyrate supplementation may not be an effective treatment option for IBD, regardless of age. Facchin et al. (2020) performed a randomized controlled trial to investigate the effects of oral administration of 1800 mg of sodium butyrate or a placebo per day, in conjunction with normal therapy, on changes in IBD activity and calprotectin levels. They found sodium-butyrate supplementation increases the growth of bacteria able to produce SCFA with potentially anti-inflammatory action.

A study conducted by Rotondo-Trivette et al. (2021) revealed significant findings indicating that Hispanic children diagnosed with UC had lower levels of fecal SCFAs than non-Hispanic children with UC, who were comparable in terms of age, sex, and illness severity. Significantly, this study had comparable pediatric UC activity index (PUCAI) scores, which allowed for an accurate overall comparison. Nevertheless, despite Hispanic children with UC displaying higher PUCAI scores, which suggest more severe disease activity, they strangely had lower fecal contents of acetate, butyrate, isovaleric acid, and propionate. Conversely, when they had lower PUCAI scores, indicating more moderate illness, they had higher fecal levels of these SCFAs.

Microbiota-derived SCFAs present a fascinating avenue for research into promoting anti-inflammatory activity in pediatric populations. As our understanding of the intricate interplay between the gut microbiota, SCFAs, and the developing immune system deepens, there is promise for novel therapeutic approaches that harness these natural compounds to foster immune resilience and mitigate inflammatory disorders in children.

### 5.2 Metabolic disorders

Microbiota-derived SCFAs stand at the intersection of the gut microbiota and pediatric metabolic health. Recent studies have shown that butyrate potentially regulates lipid metabolism, enhances insulin sensitivity, and regulates energy balance (Coppola et al., 2021). Research has shown that a deficiency in butyrate metabolism may have adverse effects on human metabolism, and such a deficiency has been observed in individuals who are obese (Magne et al., 2020; Thorburn et al., 2014). In the Butyrate Against Pediatric Obesity (BAPO) study, a six-month butyrate supplementation regimen was shown to lead to a reduction in body mass index (BMI) and improvements in inflammation and glucose metabolism (Coppola et al., 2022). In the case of thin animals and individuals, butyrate administration has been observed to enhance insulin sensitivity, thereby reducing insulin resistance and hyperinsulinemia (Matheus et al., 2017; Mattace Raso et al., 2013; Bouter et al., 2018). In individuals with diabetes, supplementation with butyrate led to a decrease in homeostatic model assessment-insulin resistance (HOMA-IR) values (Roshanravan et al., 2017). In addition, Coppola et al. (2022) have revealed that butyrate supplementation also reduces HOMA-IR and fasting insulin levels in obese children. Furthermore, a gut microbiome study provided further evidence of the role of butyrate in glucose metabolism, especially in children with higher initial concentrations of butyrate-producing bacteria, which exhibit a more favorable response (Coppola et al., 2022). In alignment with the findings of previous studies (Wang et al., 2020; Zhao et al., 2017), Coppola et al. (2022) observed a substantial reduction in waist circumference in obese children, likely attributed to the lipolytic effects of butyrate. These outcomes underscore the potential of oral butyrate supplementation to lower BMI in obese children while also exerting favorable influences on inflammation and glucose metabolism.

Prentice et al. (2019) found the presence of SCFAs such as butyrate, formic acid, and acetate in human milk, and they found that these SCFAs generally had unfavorable associations with markers of newborn obesity. However, when mixed feeding was initiated between the ages of 12 and 24 months, these effects seemed to become less pronounced. The most significant impact of these SCFAs was observed between the ages of 3 and 12 months. Therefore, the research conducted by Prentice et al. (2019) provides preliminary data indicating that the quantities of SCFAs in breast milk may provide initial protection against excessive weight gain in newborns. Dietary supplementation with butyrate has been shown to ameliorate metabolic consequences such as obesity-related inflammation, insulin resistance, and weight gain induced by high-fat diets (Mattace Raso et al., 2013; Arnoldussen et al., 2017; Vinolo et al., 2012). Interestingly, Prentice et al. (2019) suggested a potential role for butyrate derived from human milk in epigenetically influencing adipose tissue function. The study suggested that SCFAs, particularly butyrate, formic acid, and acetate, in human breast milk may be beneficial in regulating infant weight gain and adiposity. The negative association between butyrate levels in human milk and infant adiposity suggests a potential role for butyrate in negatively regulating weight gain and adipose tissue development during infancy (Prentice et al., 2019). Butyrate may influence infants’ energy metabolism, appetite regulation, or other metabolic processes, contributing to healthier growth patterns. Additionally, acetate may play a role in infants’ appetite regulation or energy balance (Prentice et al., 2019). Butyrate is known to function as an epigenetic regulator, and its involvement may influence the programming of healthy weight development in children (Davie, 2003). Alternatively, acetate might directly impact central appetite regulation, as a theory suggests (Frost et al., 2014). These findings underscore the multifaceted role of SCFAs, including butyrate and acetate, in regulating body weight and metabolic health. Therefore, while the exact underlying mechanisms remain unclear, butyrate derived from human milk potentially contributes to regulating obesity and associated metabolic changes, complementing endogenously produced butyrate.

Propionate has been shown to influence liver fatty degeneration and hasten liver damage in rats by inhibiting the oxidation of hepatocytes and disrupting oxidative metabolism in intact hepatocytes (Al-Daihan and Shafi Bhat, 2015; Glasgow and Chase, 1976). Numerous animal studies have demonstrated that maternal high-fat diet consumption leads to increased hepatic inflammation and fatty liver in fetuses and offspring (McCurdy et al., 2009; Kislal et al., 2020). The transfer of microorganisms acquired from mothers to children may be facilitated by inflammatory cytokines (Gohir et al., 2015). Therefore, reducing maternal inflammation could be a viable target for mitigating adverse metabolic outcomes in fetuses (Heerwagen et al., 2013). Research has demonstrated that butyrate therapy significantly reduces inflammatory transcription, while a high-fat diet substantially increases hepatic IL-6 levels (Mattace Raso et al., 2013). Furthermore, studies have indicated that fatty livers exhibit upregulated expression of inflammatory mediators such as TNF-α (Heerwagen et al., 2013; Li et al., 2016). Huang et al. (2022) observed increased inflammation in the fetal liver and ileum following maternal consumption of a high-fat diet, as evidenced by elevated IL-6, TNF-α, and IL-6 expression levels. However, maternal supplementation with butyrate led to a reduction in this inflammation. In a separate mouse investigation, intestinal IL-6 expression was found to be positively correlated with intestinal permeability, and a high-fat diet was associated with reduced intestinal villus length and altered epithelial barrier function (Suzuki et al., 2011; Xie et al., 2020). As our understanding of these complex interactions deepens, there is hope for developing targeted interventions that harness the potential of SCFAs to prevent and manage metabolic disorders in children, promoting a foundation for lifelong health and well-being.

### 5.3 Nervous system disorders

The early years of life are characterized by rapid neurodevelopment, and disruptions in this process can contribute to various pediatric nervous system disorders (Gilmore et al., 2018). SCFAs have promoted neurogenesis, synaptogenesis, and myelination (Mirzaei et al., 2021). Understanding how these microbial metabolites influence critical neurodevelopmental processes in children is essential for revealing potential links to neurological disorders. In this regard, Jaworska et al. (2017) investigated how microglial and astrocytic cells respond to sodium butyrate therapy. Additionally, they explored the impact of sodium butyrate on various factors, including cytokines, transcription factors, heat shock protein 70 (Hsp70), and pro- and antiapoptotic proteins. Their primary finding was that the administration of sodium butyrate has neuroprotective effects on a neonatal hypoxia-ischemia model. The efficacy of sodium butyrate in providing protection was demonstrated through a significant reduction in brain damage, inhibition of brain edema, and preservation of brain structure when assessed six days after hypoxia-ischemia onset (Jaworska et al., 2017). These results align with earlier research indicating that deacetylase inhibitors (such as valproate, trichostatin A (TSA), and sodium butyrate) have neuroprotective effects on adult rodent models of brain injury (Kim et al., 2009; Ren et al., 2004). Jaworska et al. (2017) also conducted a concise study showing the neuroprotective effects of valproate treatment after hypoxic-ischemic injury in neonatal rats. Furthermore, the administration of sodium butyrate therapy to neonates with hypoxic-ischemic injury unexpectedly led to a notable increase in the quantity of CD68 (ED1)-positive cells, specifically microglia/macrophages, in the affected hemisphere six days after insult compared to that in animals that received vehicle treatment. Notably, many of these ED1^+^ cells exhibited a favorable response to arginase-1, a well-established marker associated with the M2 microglial phenotype (Kim et al., 2009). This response was particularly pronounced in rats treated with sodium butyrate. Sodium butyrate may promote the conversion of M1 microglia to M2 microglia, subsequently triggering anti-inflammatory signaling (Kim et al., 2009). This mechanism may prevent microglia from adopting a proinflammatory phenotype and thus alleviate the tissue damage observed in models of Alzheimer’s disease (AD), multiple sclerosis (MS), and neurodegeneration (Cipriani et al., 2011; Koning et al., 2007; Jiang et al., 2021). In the present study, Jaworska et al. (2017) assessed the impact of sodium butyrate administration on the overall levels of specific cytokines associated with brain injury. The authors demonstrated significant alterations in the levels of IL-1α, IL-1β, TNF-α, and the chemokine CXC chemokine ligand 10 (CXCL10) in the hemisphere affected by hypoxic-ischemic injury 48 hours after the event compared to those in the unaffected hemisphere.

Ziemka-Nalecz et al. (2017) demonstrated that administering sodium butyrate following hypoxic-ischemic injury exerts neuroprotective effects. The authors showed that HDACis have a neuroprotective impact, as evidenced by a reduction in cerebral injury observed 14 days after hypoxia-ischemia initiation. Ziemka-Nalecz et al. (2017) provided evidence that, regardless of the extent of brain pathology caused by newborn hypoxic-ischemic injury, the injection of sodium butyrate over a five-day period following the commencement of the insult seems to mitigate cerebral damage, preventing significant atrophy or brain asymmetry. One of the findings in the present study was that the administration of sodium butyrate had a protective effect on oligodendrocyte progenitors in the ipsilateral hemisphere two weeks after the insult. This protective effect was correlated with a decrease in the number of microglia induced by hypoxic-ischemic injury and a reduction in the infiltration of macrophages/monocytes expressing the ED1 marker.

Multiple studies have established a connection between disruptions in the microbiota composition, driven by abnormalities in bacterial metabolites, and the dysregulation of immunological responses and changes in the gut-brain axis (Mirzaei et al., 2021). Several studies have shown increased permeability in the intestinal tract and various gastrointestinal abnormalities, such as dysmotility, in individuals with these disorders (Boukthir et al., 2010; De Magistris et al., 2010). Emanuele et al. (2010) found the presence of LPS and elevated levels of pro-inflammatory cytokines in individuals with ASD. Additionally, several studies have reported gut bacterial dysbiosis and alterations in the microbiota composition in ASD patients (Hsiao et al., 2013; Finegold et al., 2010; Adams et al., 2011).

Propionate is a byproduct of fermentation produced by certain bacteria, including *Desulfovibrio*, *Clostridia*, and *Bacteroidetes*. These bacteria are more abundant in the stool of individuals diagnosed with ASD (Finegold et al., 2010; Finegold et al., 2002). The observed bacterial dysbiosis in ASD patients is mostly attributed to the substantial elevation in propionate levels detected in their stool (Wang et al., 2014). Kang et al. (2013) showed that the presence and intensity of autistic symptoms were notably influenced by reduced levels of propionate-producing bacteria, specifically *Prevotella*, *Coprococcus*, and unidentified *Veillonellaceae*. Therefore, the study by Kang et al. (2013) rejects the hypothesis that high propionate levels are the only underlying factor for the abnormalities observed in all children with ASD. However, findings from other studies indicate that elevated levels of SCFAs may contribute to the development of several pathological characteristics in children with ASD (Morris et al., 2017).

Evidence from studies on ASD children indicates that they consume certain food items, such as dairy products and processed wheat, which include propionate as a food preservative (Shultz et al., 2009; Horvath et al., 1999; Jyonouchi et al., 2002). Consuming propionate-containing foods may have potential consequences, but they can vary depending on individual tolerance levels and overall dietary habits (Killingsworth et al., 2020). It’s important to note that while propionate is generally considered safe for most people when consumed in moderate amounts, excessive intake of processed foods containing this preservative may contribute to an imbalanced diet high in unhealthy fats, sugars, and sodium.

Children who are on valproate for the treatment of epilepsy may exhibit elevated levels of SCFAs, such as propionate (Horvath et al., 1999; Jyonouchi et al., 2002). Research has indicated that exposure to valproate during early development increases the likelihood of ASD (Arndt et al., 2005; Schulpis et al., 2001). Moreover, individuals with ASD who exhibit gastrointestinal symptoms and behavioral reflux have been observed to have elevated levels of *Clostridia*. The initial colonization of gut bacteria in these individuals is associated with the production of SCFAs, specifically propionate (Song et al., 2004). Furthermore, research conducted on persons with ASD has revealed evidence of metabolic dysfunction, including abnormalities in glutathione, vitamin B12, and carnitine metabolism, attributed to the impact of propionate on cellular metabolism (Wajner et al., 2004; James et al., 2006). Therefore, Shultz et al. (2009) proposed that altering the rate of metabolism of propionate may contribute to certain indicators of ASD. The authors found that the administration of propionate through intracerebroventricular injections resulted in hyperactivity, monotonous behaviors, turning behavior, caudate spiking, retropulsion, impaired social behavior, kindled seizures, heightened oxidative stress markers, and activation of an innate neuroinflammatory reaction (Shultz et al., 2009). Previous research has demonstrated that the utilization of propionate or 3-nitropropionate in animal models of propionic acidemia or Huntington’s disease has resulted in the identification of brain markers and behavioral indicators that resemble specific symptoms and characteristics associated with ASDs in humans (Brusque et al., 1999; Shear et al., 2000). As research progresses, revealing the potential therapeutic applications of SCFAs may lead to innovative strategies for preventing and managing various pediatric nervous system disorders and promoting optimal neurological health beginning in early childhood.

### 5.4 Infection

**FIGURE 2:**
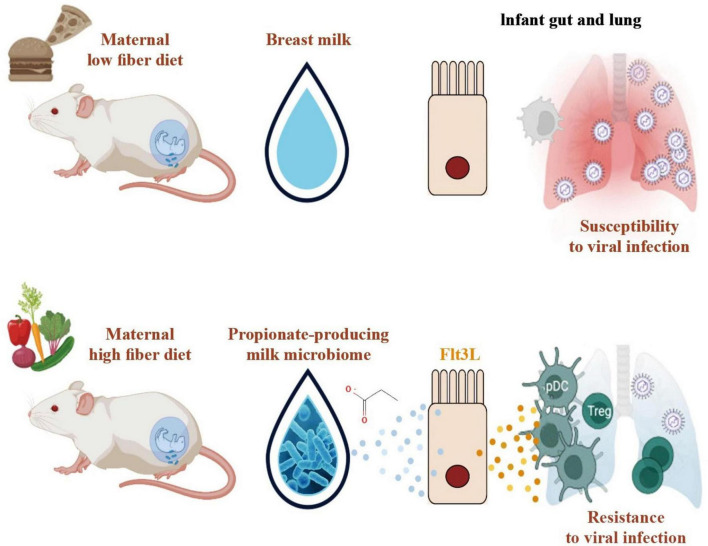
Function of microbiota-derived propionate in dendritic cell development and immunity to respiratory infection. Severe lower respiratory tract infections, a leading cause of childhood mortality, are significantly influenced by maternal diet. In this regard, a crucial link has been found between maternal dietary choices, microbiota composition, and infant immunity. Specifically, the findings emphasize the role of microbiota-derived propionate in driving dendritic cell development, fostering regulatory T-cell (Treg) expansion, and enhancing resistance to respiratory infections during the critical early stages of life. A high-fiber maternal diet promotes the development of a specific gut microbiota profile in infants. This microbiota transiently induces Fms-related tyrosine kinase 3 ligand (Flt3L), a pivotal factor in dendritic cell hematopoiesis. Dendritic cells (DCs) are essential players in the immune system and act as crucial antigen-presenting cells that initiate and regulate immune responses. These findings highlight the fact that the microbiota-driven increase in Flt3L promotes the generation and maturation of dendritic cells in neonates. The increase in the dendritic cell population, influenced by microbiota-derived propionate and Flt3L, subsequently leads to the expansion of regulatory T cells (Tregs) in the infant immune system. Tregs play a pivotal role in maintaining immune homeostasis and suppressing excessive immune responses. This orchestrated interaction between dendritic cells and Tregs contributes to improved disease resistance during early life, particularly against respiratory infections.

Severe lower respiratory tract infections (sLRIs), including conditions such as bronchiolitis and pneumonia, have a significant global impact on childhood mortality and are a leading cause of hospitalization among newborns in high-income countries (Sikder et al., 2023). Early occurrence of respiratory tract infections (LRIs) and their subsequent recurrence markedly influence lung development and physiological function (Sikder et al., 2023). Disruptions in the composition and development of the microbiota during early life have been associated with the severity of lower respiratory tract infections and the onset of chronic inflammatory diseases that begin in childhood. Sikder et al. (2023) found evidence supporting the hypothesis that a maternal diet characterized by low fiber intake during pregnancy and the subsequent preweaning phase heightens offspring’s vulnerability to sLRIs during the early postnatal period, a period known for increased risk. This vulnerability is believed to result from disturbances in the influence of the microbiota on the development and maturation of dendritic cells (DCs) in newborns. These effects are associated with fluctuations in the production of the essential growth factor Fms-like tyrosine kinase 3 ligand (Flt3L) (Sikder et al., 2023). These fluctuations are influenced by metabolites produced by microorganisms, which can stimulate significant levels of Flt3L synthesis in the IECs of the infant’s gut. Sikder et al. (2023) revealed an important pathway through which maternal microbiota affects the balance of plasmacytoid DCs (pDCs) and Tregs in newborns, impacting their susceptibility to respiratory illnesses during early infancy (Figure 2). Flt3L-mediated activation of IECs is induced by propionate, while enzymatically cleaved by ADAM10 (a disintegrin and metalloproteinase domain-containing protein 10) to facilitate this process. Once cleaved, Flt3L is released into the circulatory system, where it plays a pivotal role in DC differentiation and tissue DC maturation, priming the immune system for surveillance and protection against potential threats to the host. Disruptions in the coordinated temporal pattern of Flt3L production during development significantly affect the balance between pDCs and Tregs, increasing the vulnerability of newborns to sLRIs and the subsequent development of immunopathology associated with asthma. Increased SCFAs in the gastrointestinal system due to dietary fiber fermentation are linked to increased Treg cells in the intestinal region (Sikder et al., 2023). This occurs through the accumulation of thymus (t) Treg cells and local differentiation of peripheral (p) Treg cells, partially aided by increased transforming growth factor-β (TGF-β) expression by IECs.

In summary, these findings highlight the role of Flt3L production in IECs as a novel mechanism active during early development. This mechanism plays a critical role in maintaining DC and Treg homeostasis while reducing susceptibility to sLRIs.

A series of studies has contributed new insights into the role of gut microbiota-derived acetate in regulating the severity of lower respiratory tract diseases associated with respiratory syncytial virus (RSV) (Antunes et al., 2022). While some studies have previously demonstrated that SCFA-acetate provides defense against RSV through a GPR43- and interferon-α/β receptor (IFNAR)-dependent mechanism (Antunes et al., 2019), Antunes et al. (2022) found that SCFAs had protective effects against various RSV viral strains and on samples collected from children diagnosed with bronchiolitis, and reported the role of retinoic acid-inducible gene I (RIG-I) in modulating the antiviral response induced by SCFA-acetate. These findings also underscore the importance of the direct antiviral actions of SCFA-acetate within the respiratory tract and its potential for postinfection treatment. Additionally, evidence of the significance of acetate in preventing subsequent lung pneumococcal superinfections following influenza infection in mice has been found (Sencio et al., 2020). Acetate also offers protection against lung infections caused by *Klebsiella pneumoniae* (Galvão et al., 2018). In an *in vitro* model using human pulmonary epithelial cells, pretreatment with acetate demonstrated clear protective effects against infection with clinical RSV isolates. Antunes et al. (2022) elucidated the molecular basis for the protective effect of acetate against RSV infection by utilizing RIG-I-deficient human lung epithelial cells. According to the findings of Antunes et al. (2022), RIG-I was found to be a significant *IFN-stimulated gene* (ISG) during acetate treatment. This is attributed to the reduced impact of acetate on RIG-I expression in the absence of IFNAR1. Current research suggests that RIG-I also has a direct antiviral effect, as evidenced by the greater extent of virus replication in RIG-I-deficient cells than in cells lacking IFNAR1. While other ISGs, such as oligoadenylate synthetase 1 (OAS1), have been shown to regulate RSV replication directly, the results of the present study further support the notion that RIG-I has a direct antiviral function. The results revealed that the mice administered acetate exhibited a notable improvement in their recovery rate, coupled with a significant reduction in the viral load of RSV in the lungs. Furthermore, ex vivo experiments using respiratory epithelial cells from individuals infected with RSV via nasopharyngeal aspirates demonstrated that applying acetate to RSV-infected cells decreased RSV load and increased the expression of ISGs. In an investigation of infants admitted to the hospital with RSV bronchiolitis, Antunes et al. (2022) revealed a correlation between specific bacterial strains present in the gut microbiota and varying levels of SCFAs, including acetate. Moreover, they observed that higher acetate concentrations in fecal samples were associated with improved clinical outcomes, including elevated oxygen saturation levels upon hospital admission and a shorter duration of fever. Overall, these findings suggest that the mechanism of action of acetate involves modifying RIG-I expression, leading to a significant reduction in the viral load of RSV. Notably, in pediatric populations where the immune system is still developing, understanding how SCFAs contribute to the modulation of immune cells is pivotal for preventing and managing infections.

### 5.5 Allergic diseases

Recently, metabolomic analysis has been emphasized, especially concerning the intestinal tract. This heightened focus is due to recognizing the substantial impact of substances such as SCFAs and other microbial metabolites present in the gut on the host’s immune responses (Ito et al., 2023). These immune responses are pivotal for maintaining the equilibrium and stability of the intestinal system. Any disruption or imbalance in these immune responses can lead to the emergence of various diseases. Among these microbial metabolites, propionate is a noteworthy byproduct of microbial fermentation in the gastrointestinal tract of various animals, including humans (Ito et al., 2023). Its health-enhancing effects extend beyond the gut, including reducing cardiac hypertrophy, fibrosis, and vascular dysfunction and ameliorating colonic inflammation. These findings suggest that propionate in the gastrointestinal tract can influence the development and progression of systemic disorders and immune responses specific to the gut (Ito et al., 2023). Ito et al. (2023) involved conducting experiments on mice, aiming to demonstrate that the intake of propionate through breast milk during lactation can potentially alleviate airway inflammation in offspring within an asthma model. This effect was achieved by inhibiting eosinophil function through the signaling pathway mediated by GPR41 (Ito et al., 2023). Ito et al. (2023) showed that the administration of propionate during lactation in a mouse model of house dust mite (HDM)-induced allergic airway inflammation resulted in a reduction in eosinophilic airway inflammation and a decrease in the production of Th2 cytokines. Furthermore, they found that the effects of propionate administration were facilitated by GPR41 on eosinophils, possibly through the enhancement of TLR2, TLR8, and/or TLR9. Finally, Ito et al. (2023) A study involving a cohort of newborns revealed that the level of fecal propionate decreased at one month in individuals who later developed asthma. Trompette et al. (2014) previously reported the relationship between propionate and allergic airway inflammation. Their study demonstrated that propionate treatment in adult mice rapidly protects against acute airway inflammation, and this protective effect is dependent on GPR41. In contrast, Ito et al. (2023) revealed a novel temporal dimension of the propionate effect. Specifically, they observed that the administration of intestinal propionate during the neonatal period can effectively mitigate the development of airway inflammation in adult mice. In summary, propionate intake during the lactation period significantly reduced airway inflammation in a murine house dust mite-induced asthma model. Propionate-fed mice exhibited fewer eosinophils and less CD4^+^ T-cell exudation in the airways, reduced infiltration of eosinophils in the lung, and decreased production of the Th2 cytokines IL-5 and IL-13 (Ito et al., 2023). These findings suggest that increased intestinal propionate intake in early infancy attenuates the development of allergic airway inflammation. GPR41, but not GPR43, acts as a propionate receptor to protect against HDM-induced allergic airway inflammation in mice. A study involving GPR41 knockout (KO) mice revealed that GPR41 deficiency eliminated propionate-induced suppression of allergic airway inflammation. GPR41-expressing eosinophils were identified as the main target of ingested propionate. Propionate treatment facilitated TLR signaling in intestinal eosinophils and affected lung eosinophils through GPR41. The expression of TLR family genes was upregulated in the intestinal eosinophils of offspring born to mice fed propionate (Ito et al., 2023). These findings may have implications for the prevention of bronchial asthma in human infants. Ito et al. (2023) These findings further expand upon the findings of Trompette et al. (2014) by demonstrating the significance of GPR41 expression in eosinophils for mitigating airway inflammation through the activation of TLRs by propionate.

BPD is a chronic respiratory condition frequently observed in premature infants and is often characterized by delayed pulmonary hypertension, persistent pulmonary dysfunction, developmental delay, and neurocognitive impairments later in life (Abman et al., 2017; Davidson and Berkelhamer, 2017). Zhang et al. (2021) revealed that the administration of acetate led to a reduction in the expression levels of Nucleotide-binding and oligomerization domain (NOD)-, Leucine-rich repeat (LRR)- and pyrin domain-containing protein 3 (NLRP3)-related proteins. Additionally, acetate mitigated lung inflammation and contributed to the improvement of alveolar structure. Moreover, acetate administration has decreased NLRP3 activation through ubiquitination and autophagy, as observed in models of peritonitis and endotoxemia, signifying its anti-inflammatory properties (Xu et al., 2019). Zhang et al. (2021) also revealed that during a 14-day period of hyperoxia exposure, there was a noticeable decrease in the expression of GPR43 in neonatal mice. However, this decrease in GPR43 expression was alleviated by acetate therapy. Previous research has established a connection between the gut microbiota and the immune response in the lungs, suggesting that the gut microbiota may impact the severity of BPD by regulating systemic and lung inflammation (Lal et al., 2016; McAleer and Kolls, 2018). Since the LPS produced by *Escherichia/Shigella* can stimulate the immune system and trigger an inflammatory response, Zhang et al. reported a greater relative abundance of *Escherichia/Shigella* in the hyperoxia group than in the acetate group (Zhang et al., 2021). The study suggested a possible occurrence of heightened inflammation in the hyperoxia group. The abundance of *Ruminococcaceae*, a key producer of SCFAs, decreased following exposure to hyperoxia. However, after acetate administration, the abundance of *Ruminococcaceae* increased. These findings align with prior research indicating that *Ruminococcaceae* possesses anti-inflammatory properties and can potentially mitigate lung injury (Chua et al., 2018; Abell et al., 2008; Surendran Nair et al., 2019). These results suggest that acetate treatment holds promise as a therapeutic intervention or preventive measure for BPD.

Although there have been suggestions regarding the potential impact of infant nutrition on the subsequent development of allergies in childhood, there is still a need for effective treatments aimed at preventing allergies through dietary interventions in infants (Roduit et al., 2019). Roduit et al. (2019) hypothesized that SCFAs might play a role in mediating certain protective effects associated with the introduction of specific foods during early life. Their findings revealed a correlation between the introduction of certain foods, such as yogurt, fish, fruit, and vegetables, into an infant’s diet within the first year of life and an increase in fecal levels of SCFAs, specifically butyrate. Furthermore, children with elevated concentrations of butyrate or propionate at the age of one were less likely to be sensitized to food and/or inhalant allergens by the time they reached six years of age. Those with the highest levels of butyrate exhibited a notable tendency toward reduced susceptibility to asthma, allergic rhinitis, and food allergies. In mouse models, Roduit et al. (2019) reported reduced allergic airway inflammation with the dietary administration of butyrate, propionate, or acetate. Their investigation demonstrated that children with higher levels of SCFAs, particularly butyrate and propionate, had a significant reduction in susceptibility to atopic disorders at the age of 6 years.

**FIGURE 3:**
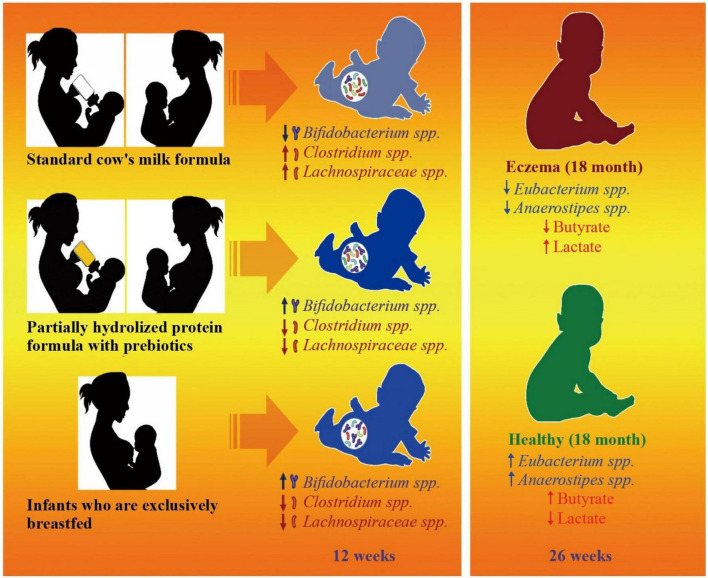
Effects of microbiota-derived short-chain fatty acids in eczema prevention and development. Eczema, a prevalent allergic skin condition, has long been associated with disruptions in the gut microbiota composition. Recent research has explored the intricate relationship between microbiota-derived short-chain fatty acids (SCFAs) and eczema prevention and development. Comparative analysis of the fecal microbial composition revealed that infants receiving the specialized formula exhibited a microbiota profile closer to that of breastfed infants, characterized by diversity and health-promoting microbial genera. Metabolite analysis demonstrated alterations in the production of SCFAs in infants with eczema compared to those without eczema. Notably, decreased butyrate levels and elevated lactate concentrations are observed in infants who develop eczema. Microbial activity, particularly the acquisition of lactate-utilizing bacteria such as *Eubacterium* and *Anaerostipes* species, is significantly reduced in infants with eczema, underscoring potential associations between microbial dysfunctions and eczema onset.

Breastfeeding has been shown to protect against the onset of allergies due to the presence of allergens and immune mediators in human milk that are not found in artificial milk (Verhasselt, 2010). Additionally, human milk oligosaccharides stimulate the development of a gut microbiota that may promote the induction of tolerance. Wopereis et al. (2018) conducted a study wherein they discovered that the introduction of a partially hydrolyzed protein formula supplemented with a specific oligosaccharide mixture (pHF-OS) can influence the maturation of the gut microbiota in infants. This influence was observed in terms of bacterial taxonomic and metabolite composition, resulting in a pattern that closely resembled that of breastfed infants. One study revealed that children who were administered pHF-OS between the ages of 4 and 26 weeks had higher proportions of the *Bifidobacterium* genus (Wopereis et al., 2018). In contrast, there were reductions in the presence of *Clostridium* species and an unidentified genus of *Lachnospiraceae* compared to infants who received conventional cow’s milk formula. The observed modulations were evident in notable variations in gut physiology, characterized by a decrease in stool pH; an increase in lactate proportions; and a decrease in propionate, butyrate, isobutyrate, and isovalerate. Additionally, the study conducted by Wopereis et al. (2018) revealed that children who experienced atopic dermatitis (eczema) within the first 18 months of life exhibited abnormal gut microbiota development during the first 26 weeks of life. Notably, substantial temporal variations were observed in the abundance of the genus *Parabacteroides* and two genera of *Enterobacteriaceae*. A decrease in these factors was observed in healthy infants over time, but infants with eczema exhibited a reversal or less pronounced manifestation of this trend. Furthermore, as time progressed, infants diagnosed with eczema exhibited diminished development of certain types of gut bacteria, specifically Eubacterium and Anaerostipes species. This finding was substantiated by a notable increase in lactate levels and a decrease in butyrate concentrations in fecal samples at 26 weeks of age (Figure 3). In summary, this study provides further evidence supporting the impact of an early-stage diet on the development of the infant gut microbiota. Furthermore, these findings suggest a possible correlation between microbial activity and the manifestation of eczema during infancy. However, further research is needed to explore the potential of increased exposure to butyrate or propionate, either through dietary intake or increased consumption of fibers fermented by the microbiota to release these SCFAs in the body, as novel strategies for allergy prevention in human clinical studies.

### 5.6 Neonatal-associated enterocolitis

In neonatal intensive care units, necrotizing enterocolitis (NEC) is a highly severe and life-threatening gastrointestinal disorder that often manifests abruptly and with great swiftness (Ferraris et al., 2023). An elevated presence of opportunistic bacterial pathogens, mainly *Enterobacteriaceae* and *Clostridium*, has been linked to the altered gut microbiota in neonates afflicted with NEC, distinguishing them from their healthy counterparts (Coggins et al., 2015; Sim et al., 2015). Recent studies have indicated that the fragile intestinal lining of newborns can suffer harm from excessive or irregular luminal concentrations of SCFAs, notably butyrate, which may surpass their natural tolerance levels (Kien et al., 1996). Ferraris et al. (2023) revealed a significant link between the fermentation of carbohydrates by bacteria and the onset of NEC. Initially, they successfully deactivated the *hbd* gene in the *Clostridium butyricum* CB1002 and *Clostridium neonatale* 250.09 strains using precise mutagenesis via homologous recombination. Both strains were originally isolated from the fecal samples of NEC patients (Waligora-Dupriet et al., 2005; Butel et al., 1998; Alfa et al., 2002). The *hbd* gene encodes a 3-hydroxy butyryl-CoA dehydrogenase that is responsible for converting acetoacetyl-CoA into 3-hydroxybutyryl-CoA, a critical step in the production of end-fermentation byproducts, such as butyrate, during the early stages of n-butanol biosynthesis from acetyl-CoA (Lo et al., 2022). In a recent investigation, Ferraris et al. (2023) observed that quails fed lactose-free diets exhibited minimal butyrate production and were free from digestive lesions. These findings underscore the metabolic role of gut bacteria in the pathogenesis of NEC, emphasizing the significance of butyrate production by *C. butyricum* and *C. neonatale* from lactose, along with their enteropathogenic effects. Notably, animals colonized with KO strains displayed increased levels of specific individual SCFAs, such as acetate, propionate, and/or branched-long-chain monomethyl fatty acids (BLCFAs), even when total cecal SCFA levels were comparable in both groups (Ferraris et al., 2023).

Sun et al. (2021) found that early intervention with sodium butyrate can effectively reduce inflammation and partially rebalance the disrupted gut microbiota in an animal model of NEC. Specifically, the administration of butyrate led to a significant decrease in the expression levels of high mobility group box 1 (HMGB1), TLR4, and NF-κB in mice with NEC. Moreover, the concentrations of proinflammatory cytokines such as IL-1β, IL-6, IL-8, and TNF-α significantly decreased, while the levels of the anti-inflammatory cytokine IL-10 notably increased following butyrate administration. Additionally, Sun et al. (2021) observed a modest decrease in the relative abundance of *Clostridium_sensu_stricto_1* following butyrate treatment. Furthermore, in the butyrate-treated group, there was a tendency for a decrease in the population of the pathogenic bacterium *Enterococcus* coupled with an increase in the population of the beneficial bacterium *Lactobacillus*. These findings indicate that butyrate administration modulates the composition of the intestinal flora, promoting the presence of beneficial *Lactobacillus* while reducing the abundance of harmful *Enterococcus*, consistent with the results reported by Li X. et al. (2021). These findings highlight the significant reduction in HMGB1 expression and proinflammatory cytokine levels and the alleviation of intestinal inflammation achieved through sodium butyrate administration.

Demehri et al. (2016) reported a significantly greater than fourfold reduction in the overall fecal levels of SCFAs in children with a history of Hirschsprung-associated enterocolitis (HAEC) compared to those who had never encountered HAEC. These findings suggested that the intestinal environment associated with HAECs is characterized by a microbial community with a reduced capacity to produce SCFAs. Given the crucial role of SCFAs in maintaining colonic mucosal integrity, this depletion of SCFAs could disrupt the balance of enterocyte function, thus contributing to the development of HAECs. Demehri et al. (2016) noted that acetate exhibited the most significant reduction in children with HAEC. Furthermore, the overall decrease in fecal SCFAs in these patients was primarily attributed to a significant decrease in acetate levels. Nevertheless, the absolute concentration of butyrate remained constant, constituting a larger proportion of the remaining pool of SCFAs. The substantial decrease in acetate levels could be due to reduced acetate production through fermentation or increased acetate absorption by the intestinal mucosa relative to enhanced acetate metabolism by colonic bacteria. The investigation conducted by Ward et al. (2012) reported the first examination of SCFA alterations in children diagnosed with Hirschsprung’s disease (HD) compared to those with a prior history of HAEC. Demehri et al. (2016) reported a substantial decrease in formate, a minor SCFA resulting from bacterial fermentation, in endothelin receptor B knock-out (KO) mice before the beginning of HAEC. There is a potential link between prior episodes of HAEC and microbiome modifications, subsequently leading to changes in SCFA levels. This relationship may contribute to the occurrence of future HAEC episodes.

In one study, Canani et al. (2013) investigated the therapeutic potential of oral butyrate in a pediatric patient with chronic liver disease (CLD). The results demonstrated a gradual reduction in the frequency and volume of bowel movements, improved stool consistency, and decreased episodes of fecal incontinence. Additionally, the study revealed a decrease in fecal electrolyte levels and the maintenance of normal serum electrolyte concentrations (Canani et al., 2004). Subsequently, Wedenoja et al. (2008) conducted a study involving five individuals with CLD who shared a homozygous frameshift mutation. Their research revealed varying responses to butyrate, suggesting that the individual’s genotype of the *SLC26A3* gene might influence the outcomes. Canani et al. (2013) substantiated the effectiveness of butyrate in regulating intestinal ion transport, particularly in a specific subgroup of CLD patients. These diverse findings underscore the multifaceted nature of the role of microbiota-derived SCFAs in NEC. Understanding the complex interactions between SCFAs, the gut microbiota, and the developing neonatal intestine holds promise for innovative preventive and therapeutic approaches to mitigate the impact of NEC and related disorders in vulnerable pediatric populations.

## 6 Microbiota short-chain fatty acids as biomarkers in pediatric diseases

As mentioned, there has been a growing recognition of the intricate relationship between gut microbiota, SCFAs, and various pediatric diseases. Liu et al. (2022) noted notable shifts in the composition of the gut microbiota and SCFAs leading to the occurrence of NEC and that SCFAs have the potential to serve as biomarkers that effectively capture the comprehensive characteristics of the gut microbiota and can be readily assessed. Notably, acetate, propionate, and butyrate levels exhibited a marked reduction, and subsequent analysis demonstrated their predictive potential before the diagnosis of NEC. In that study, the acetate, propionate, butyrate, and isovaleric acid concentrations decreased significantly in Group P (NEC group) compared with Group C (control group). The ROC curves for acetate, propionate, and butyrate between Groups C and P had AUCs of 0.73, 0.70, and 0.68, respectively. At the phylum level, certain SCFAs were negatively correlated with *Bacteroidota* and positively correlated with *Firmicutes* and *Proteobacteria*. At the genus level, specific SCFAs were positively or negatively correlated with certain genera (*Halomonas*, *Lactobacillus*, *Bacteroides*, and *Stenotrophomonas*) (Liu et al., 2022).

A study by Sun et al. (2021) further supported these findings, revealing that the introduction of butyrate mitigated intestinal inflammation and partially restored dysbacteriosis in mice with NEC. The secretion of proinflammatory factors (HMGB1, IL-1β, IL-6, IL-8, and TNF-α) was increased, and the secretion of the anti-inflammatory factor IL-10 was markedly reduced in the NEC group compared with the control and NEC + butyrate groups (Sun et al., 2021). At the phylum level, the average relative abundances of *Firmicutes* and *Bacteroidota* were greater in the NEC + butyrate subgroup than in the NEC subgroup, while the abundance of *Proteobacteria* tended to increase in the NEC subgroup (Sun et al., 2021).

Xiong et al. (2022) utilized high-throughput *16S rRNA* gene sequencing and GC-MS to perform a comparative analysis of gut microbial profiles, diversity, and metabolite characteristics in infants diagnosed with NEC and food protein-induced allergic coagulopathy (FPIAP). These findings confirmed significant differences in the fecal concentrations of SCFAs, including acetate, propionate, butyrate, iso-valeric acid, and hexanoic acid, and in the overall concentration of SCFAs between these two groups. Specifically, infants diagnosed with NEC exhibited notably lower levels of acetate, propionate, butyrate, iso-valeric acid, and total SCFAs but greater levels of hexanoic acid than infants diagnosed with FPIAP. Moreover, they explored the potential of the gut microbiota and SCFAs in the early detection of NEC and FPIAP. Xiong et al. (2022) reported area under the curve (AUC) values for acetate, propionate, butyrate, isovaleric acid, hexanoic acid, and total SCFAs of 0.8398, 0.8680, 0.8593, 0.7641, 0.7576, and 0.8658, respectively. Furthermore, the composition of the gut microbiota and SCFA concentrations in newborns with NEC differed from those in infants with FPIAP. The present study revealed a greater abundance of *Actinobacteria*, *Halomonas*, *Acinetobacter*, *Bifidobacterium*, and *Stenotrophomonas* in NEC infants than in FPIAP infants. Conversely, the abundance of *Bacteroidota* was lower in infants with NEC. Therefore, the alterations observed in acetate, propionate, and butyrate can indicate the gastrointestinal abnormalities associated with NEC and aid in early prognosis.

Certainly, discussing the differences in SCFAs under various health conditions, including production and absorption processes in normal physiological states versus disease states, is crucial for potential therapeutic approaches. In a study by Łoniewska et al. (2023) SCFA concentrations in the stool increased from the beginning of life (at which the meconium sample was collected) until 12 months of age. The acetate concentration increased significantly only up to one month after birth, with no significant increase thereafter. Propionate, butyrate, and total SCFA concentrations significantly increase over time (Łoniewska et al., 2023). The present study indicated that perinatal factors may influence changes in fecal SCFA levels. Bridgman et al. (2017) discovered that exclusively breastfed infants had reduced absolute concentrations of SCFAs. Martin et al. (2014) discovered reduced levels of SCFAs in 111 fecal samples analyzed by nuclear magnetic resonance (NMR) from mothers who were breastfed at three and six months and were delivered to mothers who were overweight or obese. Using gas chromatography-liquid chromatography (GC-LC) mass spectrometry, a small study of 4 infants determined that formula-fed infants had higher concentrations of valerate and isovalerate, with the latter being more than forty times greater than that of breastfed infants (Chow et al., 2014; Ogawa et al., 1992). An investigation involving 67 infants not only revealed reduced fecal SCFA concentrations in those who were breastfed but also noted that supplementing breastfed infants’ diets with milk (whether it be formula or cow’s milk) was adequate for altering the SCFA profile (Ogawa et al., 1992). Bridgman et al. (2017) noted comparable outcomes, whereby the SCFA and lactate concentrations of partially breastfed infants were more comparable to those of exclusively formula-fed infants than those of exclusively breastfed infants.

In disease states, gut microbiota composition and function alterations can impact SCFA production. For example, Gio-Batta et al. (2020) found that children with eczema had a lower median concentration of valeric acid in their feces at three years of age (Gio-Batta et al., 2020). Additionally, conditions such as IBD may result in changes in SCFA levels. In children diagnosed with UC, Rotondo-Trivette et al. (2021) reported the concentrations of the following SCFAs 49.52 micromolar (μM) for acetate, 28.32 μM for butyrate, 2.08 μM for formic acid, 1.79 μM for isovaleric acid, and 17.95 μM for propionate. In summary, understanding the exact concentrations of SCFAs in different states is vital for tailoring interventions to promote overall health and address specific diseases. Further research is essential to refine the use of SCFAs as diagnostic tools and to unlock their full potential in improving pediatric healthcare.

## 7 Manipulation of short-chain fatty acids production in pediatric populations via modulation of the microbiota

Modulating SCFA production through microbiota modulation has emerged as a promising avenue for enhancing pediatric health. In a study conducted by Gunawan et al. (2021), the *Bifidobacterium* genus displayed a notable level of resilience in response to changes in SCFA levels and a reduction in fecal pH. The analysis of SCFAs revealed significant increases in acetate, propionate, and butyrate concentrations in the group that consumed the symbiotic powder for 90 days. The synergistic effect of probiotics and prebiotics can decrease the intestinal lumen’s pH and stimulate cell proliferation (Gunawan et al., 2021). This, in turn, increases the surface area available for mineral absorption, enhancing mineral bioavailability and ultimately improving nutritional status. The gastrointestinal tract conveys information about nutritional status through various signals, including gut-derived hormones such as glucagon-like peptide-1 (GLP-1). Postmeal increases in GLP-1 influence metabolic processes, such as promoting insulin secretion, delaying stomach emptying, and enhancing satiety. Previous studies have shown that adding fermentable fibers to the diet can increase GLP-1 levels in both rodents and humans. Thus, SCFAs produced by the gut microbiota are hypothesized to lead to increased GLP-1 levels. Moreover, research on germ-free mice has shown that the absence of SCFAs produced by microorganisms in the colon leads to a significant increase in bloodstream levels of GLP-1. This colonic-derived GLP-1 appears to play an adaptive role in enhancing nutrient absorption by delaying transit time in the small intestine (Wichmann et al., 2013). Gunawan et al. (2021) provided evidence that a 90-day regimen of consuming a symbiotic powder containing *Lactobacillus plantarum* DAD 13 [Dadih” (traditional fermented buffalo milk)] and fructo-oligosaccharides (FOS) resulted in increased SCFA levels, specifically acetate, propionate, and butyrate, while simultaneously lowering the fecal pH.

Holmes et al. (2020) revealed that the microbiota of all examined adolescents with obesity exhibited an increase in overall SCFA synthesis upon *in vitro* exposure to at least one prebiotic. Both the donor and prebiotic were found to be influential elements influencing the formation of SCFAs *in vitro*. Additionally, their interaction was also observed to have a substantial impact on SCFA production. The modeling analysis results (Holmes et al., 2020) demonstrated clear and discernible connections between particular microbial taxa and the formation of SCFAs in response to various prebiotics. These findings can be interpreted as indicating that the presence of certain bacteria is connected with the community’s ability to ferment fiber. The current study replicated prior research indicating that both the donor and prebiotic have a significant role in influencing the generation of SCFAs through *in vitro* prebiotic supplementation (Venkataraman et al., 2016; Gamage et al., 2018).

These findings indicate that heightened signaling via GPCRs, facilitated by acetate, propionate, and butyrate, leads to enhanced feelings of satiety and improved insulin sensitivity while simultaneously reducing the process of adipogenesis (Samuel et al., 2008; Tolhurst et al., 2012; Kimura et al., 2013). In experimental settings, it has been demonstrated that elevated butyrate levels within a controlled environment can stimulate the upregulation of the transporter solute carrier family 5 member 8 (SLC5A8) (Mathewson et al., 2016). Additionally, the introduction of physiological combinations of SCFAs has been shown to induce the upregulation of the transporter solute carrier family 16 member 1 (SLC16A1) (Zhan et al., 2018). Both transporters are responsible for acetate, propionate, and butyrate uptake from the luminal space. By understanding and harnessing the intricate interplay between gut microbiota and SCFAs, we can pave the way for innovative strategies that contribute to well-being and disease prevention in the pediatric population.

## 8 Conclusion

In conclusion, the study of microbiota-derived SCFAs in children’s health and diseases represents a fascinating and evolving field of research. Over the years, significant progress has been made in understanding the intricate relationships among the gut microbiota, SCFAs, and various aspects of child health, from infant development to neuroprotection. Through extensive research, we reviewed the profound impact that SCFAs can have on the developing immune system, metabolic health, and even cognitive function in children. However, as we progress, several important future research directions emerge. First and foremost, there is a need for further elucidation of the specific mechanisms by which SCFAs exert their effects on children’s health. Understanding the specific signaling pathways and cellular interactions involved will provide valuable insights for targeted therapeutic interventions. Second, we should explore the potential for modulating the gut microbiota and SCFA production in children through dietary interventions, probiotics, or other means. This could have profound implications for preventing and managing childhood diseases and optimizing developmental outcomes. In summary, microbiota-derived SCFAs are emerging as key players in children’s health and disease status and could revolutionize our approach to pediatric healthcare. As we continue to unravel the complexities of this field, there is great promise for improving children’s well-being and developmental outcomes through targeted interventions that harness the power of SCFAs and the gut microbiota.
